# Soil Nitrogen Treatment Alters Microbiome Networks Across Farm Niches

**DOI:** 10.3389/fmicb.2021.786156

**Published:** 2022-02-14

**Authors:** XinYue Wang, Kerri Reilly, Rosemary Heathcott, Ambarish Biswas, Linda J. Johnson, Suliana Teasdale, Gwen-Aëlle Grelet, Anastasija Podolyan, Pablo Gregorini, Graeme T. Attwood, Nikola Palevich, Sergio E. Morales

**Affiliations:** ^1^Department of Microbiology and Immunology, University of Otago, Dunedin, New Zealand; ^2^AgResearch Limited, Grasslands Research Centre, Palmerston North, New Zealand; ^3^Manaaki Whenua – Landcare Research, Lincoln, New Zealand; ^4^Department of Agricultural Science, Lincoln University, Lincoln, New Zealand

**Keywords:** agriculture, microbiomes, 16S, 18S, ITS, amplicon sequencing, nitrogen treatment, microbiome networks

## Abstract

Agriculture is fundamental for food production, and microbiomes support agriculture through multiple essential ecosystem services. Despite the importance of individual (i.e., niche specific) agricultural microbiomes, microbiome interactions across niches are not well-understood. To observe the linkages between nearby agricultural microbiomes, multiple approaches (16S, 18S, and ITS) were used to inspect a broad coverage of niche microbiomes. Here we examined agricultural microbiome responses to 3 different nitrogen treatments (0, 150, and 300 kg/ha/yr) in soil and tracked linked responses in other neighbouring farm niches (rumen, faecal, white clover leaf, white clover root, rye grass leaf, and rye grass root). Nitrogen treatment had little impact on microbiome structure or composition across niches, but drastically reduced the microbiome network connectivity in soil. Networks of 16S microbiomes were the most sensitive to nitrogen treatment across amplicons, where ITS microbiome networks were the least responsive. Nitrogen enrichment in soil altered soil and the neighbouring microbiome networks, supporting our hypotheses that nitrogen treatment in soil altered microbiomes in soil and in nearby niches. This suggested that agricultural microbiomes across farm niches are ecologically interactive. Therefore, knock-on effects on neighbouring niches should be considered when management is applied to a single agricultural niche.

## Introduction

A farm is a collection of interlinked ecological habitats split by locations, including above-ground, below-ground, and animal-associated niches each harbouring unique microbiomes. Microbiomes are essential as providers of ecosystem services including biogeochemical nutrient cycling, greenhouse gas emission, and host-microbiome interactions (Kinross et al., 2011; Chaparro et al., 2012; Rubino et al., 2017). The interactions within and between niche microbiomes are the drivers for nutrient cycling through microbial metabolic processes, such as bacterial-plant symbioses and nitrogen cycling (Richardson et al., 2009; Mendes et al., 2013; Vandenkoornhuyse et al., 2015). Saprotrophic organisms in soil break down organic matter into composites inducing fertility (Anderson et al., 1981; Dighton, 2007; Morris and Blackwood, 2015). Microbiomes from phyllosphere (above-ground parts associated with plants) and rhizosphere (below-ground parts associated with roots) provide benefits to host growth and wellbeing (Adesemoye et al., 2009), for instance helping with nutrient acquisition from soil, defence against plant-pathogens or adapting to environmental stresses (Mendes et al., 2013; Turner et al., 2013; Muller et al., 2016). These benefits are also seen in animal microbiomes through nutrient acquisition mediated by gut microbiomes (Flint et al., 2008).

Physical and functional connections at a multi-niche scale are poorly understood in farm ecosystems but are established in the human microbiomes. Significant co-occurrence and co-exclusion are found between human microbiomes from different body sites, where most relationships are niche-specific but multi-niche linkages are also discovered (Faust et al., 2012). Although microbiomes interactions across multiple farm niches have not been widely established (Turner et al., 2013), it is reasonable to postulate that there are microbial interactions across multiple niches based on the accumulated evidences from different studies. For instance, change of nutrition in soils can lead to variations of vegetation (i.e., pasture) compositions in farmlands (Ledgard et al., 2001; Eschen et al., 2007). The altered vegetation compositions would lead to a diet change for grazing animals which consequently affect the rumen microbiomes.

Fertilisers are commonly used for better agricultural yields, but intensive fertilisations could also introduce environmental and biological impacts on soils and other linked habitats. Application of fertilisers supplies a large amount of nutrients which could lead to increased greenhouse gas emissions, nutrient leaching, and disturbances to microbiomes (Snyder et al., 2009; Potter et al., 2010; Wang et al., 2017). Many studies focused on fertilisation impacts on microbiomes in individual niches [e.g., soil or rhizosphere (Ramirez et al., 2012; Zeng et al., 2016; Wang et al., 2018)], but impacts on surrounding niches were unexplored. Apart from microbiome diversities and compositions, both organic and inorganic fertilisation influenced microbiome networks, consequently affected microbiome community stability (Coyte et al., 2015; Butler and O’Dwyer, 2018). Liu et al. (2020) showed that soil organic fertilisation was linked to more connective soil microbiome networks compared to that without fertilisation, but Wang et al. (2017) found that organic fertiliser treated soil had less interactions among bacteria and fungi. Overall, findings on fertilisation effects in agricultural ecosystem were complex and usually from a narrow focus (in individual niches). Thus more attention is required to investigate if and how disturbances to a single habitat have successive influences on other interlinked habitats. In other words, a broader view of treatment effects should be taken on a multi-niche scale to capture a more complete view on the entire ecosystem.

Therefore, the current study examined microbiome responses to nitrogen treatment applied to soils across different agricultural niches within the same farm. The objectives of this study were (1) to better understand microbiomes in individual farm niches, and (2) to investigate linkages between microbiomes across farm niches, using nitrogen treatment (i.e., urea based fertilisation) as disturbances. In order to obtain representational characteristics of microbiomes across farm niches (i.e., soil, ryegrass root, ryegrass leaf, white clover root white clover leaf, faecal, and rumen), multiple approaches (16S, 18S, and ITS amplicon sequencing) focusing on specific organisms were used. Alpha diversities, beta diversities, and microbiome networks analyses were used to asses both microbiomes and their linkages across farm niches. We hypothesised that (1) nitrogen treatment in soil reduces microbiomes richness, composition, or networks in soil and nearby niches in prokaryotes; (2) nitrogen treatment will have unique responses across each microbiome subpopulation (i.e., prokaryotes, eukaryotes, and fungi).

## Materials and Methods

### Farm Site and Urea Treatment

The experiments were carried out at perennial ryegrass and white clover swards at Lincoln University’s Ashley Dene Research and Development Station, Canterbury, New Zealand (−43.6468, 172.3467). Urea was applied to separate sampling sites during the farm season (1 June 2017 to 31 May 2018) to achieve designed nitrogen treatment [no-nitrogen (0 N kg/ha/yr), medium-nitrogen (150 N kg/ha/yr), and high-nitrogen (300 N kg/ha/yr)]. The three levels of nitrogen treatment were selected based on the range of nitrogen application rates in pastoral dairy farms in Canterbury as previously described (Beukes et al., 2020).

The experimental swards were grazed by 30 lactating dairy cows (10 cows per nitrogen treatment) to allow the normal grazing practise for pasture and animals. Same amount of herbage intake was controlled across cows (∼30 kg DM/cow/day) by break feeding. To stabilise the environmental microbiomes, the experimental sites were grazed by cows 2 weeks before sampling. The allocated swards were examined daily before and after grazing using a rising-plate meter (RPM) to estimate pasture intake during sampling by using the equation RPM reading ×140 + 500 kg DM/ha.

### Sample Collections

Seven types of samples (bulk soil, cow rumen content, cow faeces, white clover leaf, white clover root, ryegrass leaf, and ryegrass root) were taken from three ecological niches (soil, plant, and animals). A total of 169 samples were used in the current study where metadata details can be found in Supplementary Table 1.

Soil cores were taken using a 110 mm wide (internal measurement) corer, with a 100 mm wide (internal measurement) plastic sleeve inserted. Plant materials were gently twisted into the centre of the core and sleeve to minimise damage during sampling. The corer was driven into the soil to a depth of 150 mm to ensure that at least the top 100 mm soil is recovered intact. Once the depth was reached, the corer was gently twisted and lifted out of the ground. Two smaller plastic sleeves were used to transfer the soil from the corer. The soil sample was then wrapped in clingfilm and stored at 4°C. Bulk soil samples were taken after removing stones and plant materials.

Rumen samples were collected by stomach tubing of animals. Faecal samples were taken with digital collection from cow anuses at the same time of rumen content collection. Both types of animal samples were flash frozen with liquid nitrogen and stored at −80°C until further progress.

For both white clover and ryegrass, individual plants were gently removed from the soil core and split into root and leaf parts with a sterile scalpel blade. After removing loose soil particles, the plant samples were then snap froze with liquid nitrogen and stored at −80°C until DNA extractions.

### DNA Extractions and Amplicon Sequencing

DNA was extracted from individual sample (300 mg/sample) with a Macherey-Nagel NucleoSpin 96 Soil kit. The manufacturer’s protocol was followed but with the following modifications. Buffer SL1 (700 μL) and enhancer SX (300 μL) was pre-combined and added to a 1.7ml tube. Grounded sample was then transferred to the tube and vortexed for 1 min. The mixture was then centrifuged for 4 min at 11,000 x *g*. After removing the supernatant, 150 μL buffer SL3 was added to the tube. The tube was then incubated at 4°C for 20 min and centrifuged for 2 min. The clear supernatant was then processed following manufacture protocols and eluted twice with 70 μL of buffer SE each time.

We examined microbiomes using 3 approaches focusing on specific microbiome subpopulations, namely prokaryotes (16S rRNA), eukaryotes (18S rRNA), and fungi (ITS rRNA). Next-generation sequencing data were captured from 7 farm niches, specifically 2 animal-associated niches [Rumen (R) and Faecal (F)], 3 below-ground niches [WhiteCloverRoot (WCR), RyeGrassRoot (RGR), and Bulk Soil (BS)], and 2 above-ground niches [WhiteCloverLeaf (WCL) and RyeGrassLeaf (RGL)]. For each of these microbiomes, community responses were compared across three different soil nitrogen treatments [control or no-nitrogen (0 N kg/ha/yr), medium-nitrogen (150 N kg/ha/yr), and high-nitrogen (300 N kg/ha/yr)].

Next generation sequencing was conducted with an Illumina MiSeq V2 platform at Massey University. The total community genomic DNA for each sample was sequenced targeting 16S ribosomal RNA (rRNA), 18S rRNA, and internal transcribed spacer (ITS) regions with primers following the Earth Microbiome Project.

The demultiplexed paired end sequence reads were then quality filtered and merged using dada2 package V1.12 (Callahan et al., 2016) in R V3.6.1 (R Core Team, 2019) following the Dada2 tutorial pipelines (16S and 18S, ITS) with default settings. The merged sequence reads were then denoised, chimera removed, and dereplicated into Amplicon Sequence Variant (ASV). Silva v132 (Quast et al., 2013), and UNITE databases (Nilsson et al., 2019) were used to assign taxonomy to each ASV. For each amplicon, ASV table, metadata (Supplementary Table 1), and taxonomy table were converted into a phyloseq object with phyloseq version 1.28.0 (McMurdie and Holmes, 2013). The raw amplicon (16S, 18s, and ITS) sequencing files were uploaded to the NCBI SRA database under the accession number PRJNA702151. For all sample of each dataset (16S, 18S, and ITS) sequences were rarefied to an even depth, that 10000, 25000, and 10000 reads per sample were randomly selected for 16S, 18S, and ITS datasets, respectively. For 16S phyloseq object, chloroplasts sequences were removed. Singletons were discarded prior to from downstream diversity analyses. R scripts of all data processing were accessible online from GitHub.

### Statistical Analyses and Visualisation

Amplicon sequence variant counts for each sample across farm niches were generated based on rarefied and trimmed (i.e., singletons removed) datasets (16S, 18S, and ITS). The alpha-diversity measurements across farm niches and in response to nitrogen treatments was calculated by function *estimate_richness* using the “Observed” method in R with packages dplyr version 0.8.3 (Wickham et al., 2019), plyr version 1.8.4 (Wickham, 2011), reshape2 version 1.4.3 (Wickham, 2007), and vegan version 2.5.6 (Jari Oksanen et al., 2019). The violin plots were created with function *plot_richness* facet by niche type and ecosystem type using packages ggplot2 version 1.28.0 (Wickham, 2016), Biostrings version 2.52.0 (Pagès et al., 2019), forcats version 0.4.0 (Wickham, 2019), ggpubr version 0.2.3 (Kassambara, 2019), lemon version 0.4.3 (Edwards, 2019), phyloseq version 1.28.0 (McMurdie and Holmes, 2013), RColorBrewer version 1.1.2 (Neuwirth, 2014), and scales version 1.0.0 (Wickham, 2018). Kruskal-Wallis tests were used to compare species richness across farm niches and nitrogen treatments that statistical significances were masked on the violine plots with function *stat_compare_means* of package ggpubr version 0.2.3 (Kassambara, 2019). Microbiome dissimilarities across all samples were determined using a phyloseq function *ordinate* with the Bray-Curtis distance method (i.e., distance = “bray”). Beta-diveristy of all samples across treatments were visualised with NMDS plots using function *plot_ordination* with *stat_ellipse* option. Changes in community structure and variance were tested via functions anosim and adonis, respectively, with vegan version 2.5.6 (Jari Oksanen et al., 2019). Both functions were conducted using Bray-Curtis distance matrix as the input data matrix and N treatment (i.e., no nitrogen, medium nitrogen, and high nitrogen) as grouping.

Exact tests were used to determine differentially abundant ASVs for each individual niche between nitrogen treated and untreated microbiomes illustrated with a dot plot. For each phyloseq dataset (16S, 18S, and ITS), Packages edgeR version 3.26.8 (Robinson et al., 2010), ggplot2 version 1.28.0 (Wickham, 2016), ggpubr version 0.2.3 (Kassambara, 2019), ggrepel version 0.8.1 (Slowikowski, 2019), lemon version 0.4.3 (Edwards, 2019), and phyloseq version 1.28.0 (McMurdie and Holmes, 2013) were used. Heatmap and volcano plot were used to supplement Exact tests. Heatmaps provided the presence and absence information on responsive ASVs across individual niches between samples. Volcano plots illustrated the changing direction (positive or negative) for ASVs responses across niches and between nitrogen treatments. R packages ggplot2 version 1.28.0 (Wickham, 2016), ggpubr version 0.2.3 (Kassambara, 2019), lemon version 0.4.3 (Edwards, 2019), and phyloseq version 1.28.0 (McMurdie and Holmes, 2013) were used.

Network correlations for each niche under each nitrogen treatment were constructed with packages igraph version 1.2.4.1 (Csardi and Nepusz, 2006), networkD3 version 0.4 (Allaire et al., 2017), and tidyr version 1.0.0 (Wickham and Henry, 2019) to provide insight into the microbial interactions responses to different nitrogen managements. In each niche, ASVs were chosen based on two criteria: (1) were present in at least 60% of samples; and (2) were significantly (*p* ≤ 0.05) differentially abundant in response to nitrogen treatments in any niche, or were the top 200 most abundant across niches. Spearman correlations were conducted between all chosen ASV pairs in each niches with package psych version 1.8.12 (Revelle, 2018). ASV pairs having strong significant correlations (*r* ≥ 0.8 or *r* ≤ −0.8, *p* ≤ 0.05) were included in the networks. The force-directed layout algorithm was then used to calculated the network layout. Nodes were coloured by phylum represent ASVs and edges were coloured by correlation positiveness represent significant microbial coefficients.

Niche microbiome metapopulation was investigated by analysing occupancy-frequency distribution of ASVs in each niche was made with packages ggplot2 version 1.28.0 (Wickham, 2016), vegan version 2.5.6 (Jari Oksanen et al., 2019), and scales version 1.0.0 (Wickham, 2018) following previous study (Lindh et al., 2017). In the current study, the occupancy of an ASV is defined as the fraction of samples it occupied in each niche. The frequency denotes the existence number of unique ASVs in a certain proportion of samples in each niche.

## Results

From samples across farm niches, an average of 111588, 102169, and 98632 raw reads (16S, 18S, and ITS, respectively) per sample were generated with details listed in Supplementary Table 1. After quality control, the mean quality scores were 36.67 ± 0.14, 36.89 ± 0.26, and 35.84 ± 0.75 for 16S, 18S, and ITS reads, respectively. An average of 53748, 84857, and 56414 non-chimeric reads for 16S, 18S, and ITS reads, respectively, were obtained.

### Microbial Richness and Composition Were Distinct Across Niches, but Shared Similar Responses to Soil Nitrogen Treatment

**FIGURE 1:**
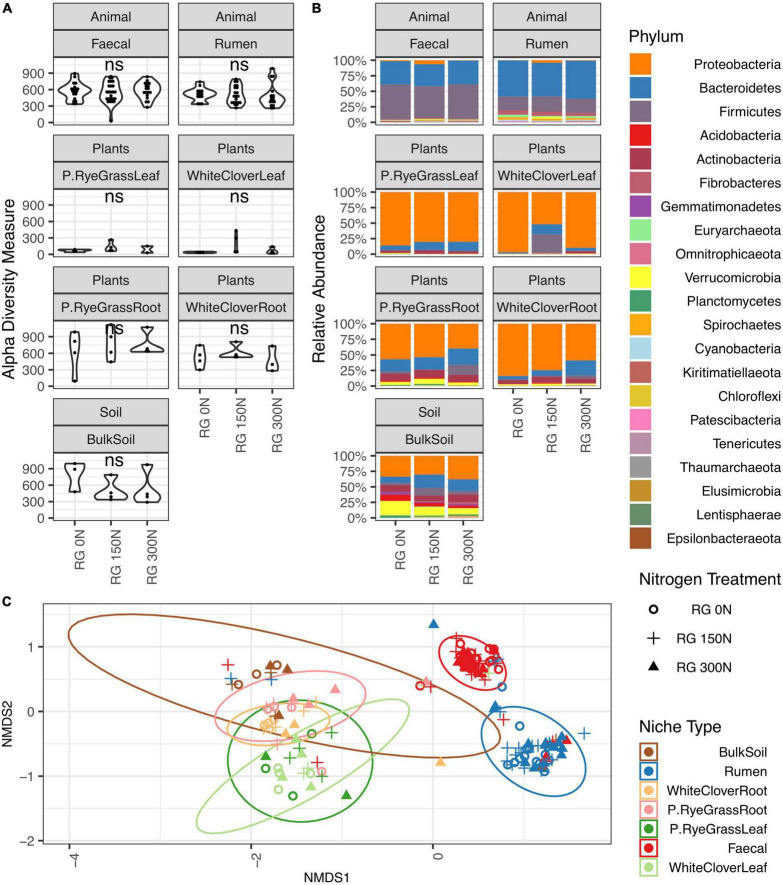
16S Microbiome diversities and composition comparisons across farm niches under three levels of nitrogen treatments (0, 150, and 300 N/ha/yr). **(A)** Microbiome richness based on number of observed ASVs are shaped by nitrogen treatments. Open circle 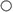, plus symbol + and closed triangle ▲ represent 0, 150, and 300 N/ha/yr nitrogen managements, respectively. Statistical significances shown in figure were calculated with Kruskal-Wallis test, where ns represents not significant (adjusted *p*-value > 0.05). **(B)** Relative abundance of microbiome taxa across farm niches coloured at Phylum level. **(C)** NMDS plot using Bray-Curtis distance coloured by niches and shaped by treatment levels. ANOSIM statistic *R*: 0.7534, significance: 0.001; ADONIS *R*^2^: 0.52944, significance: 0.001.

To compare the microbiome richness across niches and nitrogen treatment, Alpha diversities were calculated and analysed across niche microbiomes with ASVs (amplicon sequence variants). For all three amplicons, richness of microbiomes were distinct across farm niches (*p* < 0.001, Kruskal-Wallis rank sum test) (Table 1 and Supplementary Table 2), but were not significantly differentiated between nitrogen treatments (Figure 1A, Supplementary Figures 1A–2A, and Supplementary Table 2). In general, leaf microbiomes were the least diverse where BS microbiomes richness under no-nitrogen were the highest across amplicons, with an average of 788, 357, and 385 observed ASVs from 16S, 18S, and ITS, respectively. In contrast, phyllosphere microbiomes richness were relatively low. For instance WCL microbiome richness under no-nitrogen were low across amplicons, with an average of 36, 20, and 149 observed ASVs from 16S, 18S, and ITS, respectively. Nitrogen treatment did not alter microbiome richness except for ITS of below-ground niche WCR (Supplementary Figure 2A), where microbiome richness was decreased with soil nitrogen treatment (*p* = 0.045, Kruskal-Wallis rank sum test).

**TABLE 1:** Beta-diversity statistics for 16S, 18S, and ITS microbiomes.

SampleType	ANOSIM_Significance	ANOSIM_Stat_R	ADONIS_Significances	R_Square	Amplicon type
Faecal	0.81	−0.02281156	0.384	0.01721146	16S
Rumen	0.79	−0.0267907	0.694	0.01489381	16S
P.RyeGrassLeaf	0.74	−0.13076923	0.6	0.09034385	16S
WhiteCloverLeaf	0.089	0.12268519	0.397	0.08323055	16S
BulkSoil	0.948	−0.16	0.399	0.09743818	16S
P.RyeGrassRoot	0.845	−0.08101852	0.509	0.08627943	16S
**WhiteCloverRoot**	**0.004**	**0.31666667**	**0.01**	**0.20113337**	**16S**
Faecal	0.743	−0.018038478	0.23	0.03783233	ITS
Rumen	0.367	0.006750401	0.268	0.04688803	ITS
P.RyeGrassLeaf	0.796	−0.169230769	0.883	0.16767965	ITS
WhiteCloverLeaf	0.82	−0.122685185	0.717	0.14675937	ITS
BulkSoil	0.973	−0.173333333	0.803	0.15159959	ITS
P.RyeGrassRoot	0.926	−0.165509259	0.939	0.13238359	ITS
WhiteCloverRoot	0.493	−0.016666667	0.457	0.19348201	ITS
Faecal	0.321	0.03348174	0.322	0.008048333	18S
**Rumen**	**0.027**	**0.04298456**	**0.048**	**0.056111693**	**18S**
P.RyeGrassLeaf	0.923	0.08604594	0.545	−0.038461538	18S
WhiteCloverLeaf	0.826	0.17984389	0.704	−0.083333333	18S
BulkSoil	0.75	0.15431821	0.917	−0.131666667	18S
P.RyeGrassRoot	0.153	0.27937883	0.057	0.19	18S
WhiteCloverRoot	0.056	0.31870693	0.047	0.216666667	18S

*ANOSIM and Adonis (Bray-Curtis dissimilarity matrix) are used for determining nitrogen treatment effects for individual niche. Significant data are showing in bold.*

Taxonomic compositions were distinct across niches, but did not change in response to soil nitrogen treatment (Figure 1B, Supplementary Figures 1B–2B, and Supplementary Tables 3A–C). For 16S communities, regardless of nitrogen treatment, the phyllosphere microbiomes in both RGL and WCL were dominated by *Proteobacteria* (82 and 79% on average, respectively). Similarly in soil and rhizosphere niches (BS, RGR, and WCR), *Proteobacteria* dominated at 28, 36, and 47% on average, respectively. Animal-associated microbiomes R and F were both dominated by *Bacteroidetes* and *Firmicutes* (Supplementary Table 3A). Trends (i.e., changes across niches, with no treatment effects) were similar for 18S and ITS microbiomes (Supplementary Figures 1B–2B and Supplementary Tables 3B,C) with a couple of exceptions. For 18S, *Phragmoplastophyta* dominated in all phyllosphere and rhizosphere niches, while *Cilliophora* dominated the 2 animal-associated niches (Supplementary Figure 1 and Supplementary Table 3B). For ITS, *Ascomycota* dominated across all niches except for R, where *Neocallimastigomycota* was the most abundant (Supplementary Figure 2 and Supplementary Table 3C).

Bray-Curtis distances were calculated and plotted with NMDS ordinations to examine microbiome beta diversities across niches. Significant differences in community composition were observed across niche microbiomes (16S: ANOSIM: *R* = 0.758 and ADONIS: *R*^2^ = 0.505. 18S: ANOSIM: *R* = 0.246 and ADONIS: *R*^2^ = 0.248. ITS: ANOSIM: *R* = 0.647, ADONIS: *R*^2^ = 0.325, *p* < 0.01 for all cases), but not in response to nitrogen treatment (Figure 1C, Supplementary Figures 1C–2C, and Table 1) except for WCR (16S community) and R (18S community). For 16S, animal-associated microbiomes were more distinct compared with other niches (Figure 1C). Samples from the rhizosphere were clustered primarily by niches, but partial overlaps were observed between BS, RGR, RGL, WCR, and WCL microbiomes. Microbiome compositions for 18S and ITS were also significantly different across niches, but broad overlaps across niches were found. Nitrogen treatment had minimal impact on microbiome compositions within each individual niche except for 16S microbiomes in WCR (ANOSIM *p* = 0.004, *R* = 0.317 and ADONIS *p* = 0.01, *R*^2^ = 0.189), and for 18S microbiomes in R (ANOSIM *p* = 0.027, *R* = 0.043 and ADONIS *p* = 0.048, *R*^2^ = 0.056).

**FIGURE 2:**
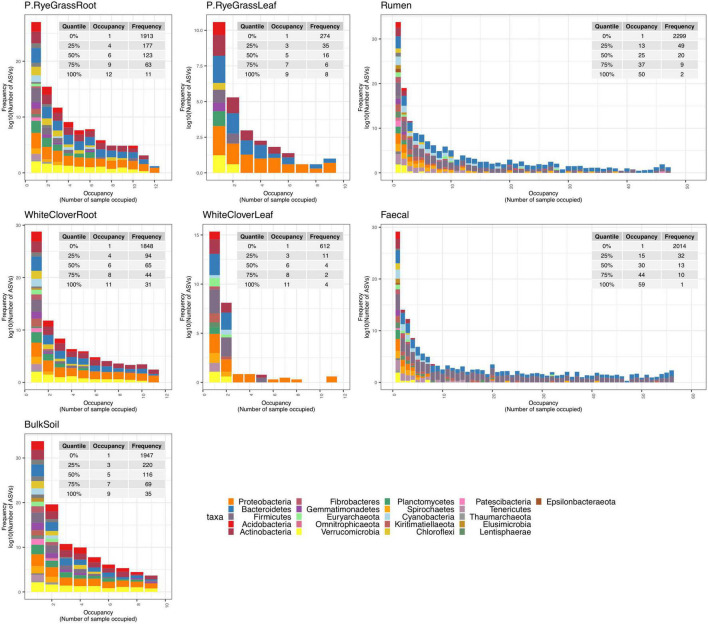
Microbiome frequency and occupancy plot (16S). Different number of populations occupying different number of samples at each niche are illustrated where *X*-axis illustrates number of sample sites panelled by each individual niche and *Y*-axis illustrates accumulated number of present ASV in certain number of samples. For example, example in panel Rumen, occupancy of 1 and frequency of 2,299 indicate there are 2,299 unique ASVs only present in a single sample out of all rumen samples, where there are two unique ASVs found in 50 rumen samples.

To compliment the microbiome compositional and structural change in response to nitrogen treatment, microbiome structural dynamics were also measured for each amplicon across niches with frequency and occupancy plots (Figure 2 and Supplementary Figures 3, 4). Microbiomes occupancy-frequency distributions across amplicons in both of the phyllosphere niches showed similar skewed patterns. The number of shared ASV declined with increasing number of samples within niche, followed by an increase in species occupying all or most sites, illustrating a core-satellite pattern (Hanski, 1982; Lindh et al., 2017). Compared to the phyllosphere microbiomes, rhizosphere microbiomes showed variations in distribution dynamics across amplicons. Core-satellite patterns were found in ITS and 18S communities, but not in 16S, suggesting variations in community assembly mechanisms and selective pressure across sub-populations and niches.

### Nitrogen Treatment Affected Amplicon Sequence Variant Abundance, but Only for Certain Taxa in Animal Associated Niches

**FIGURE 3:**
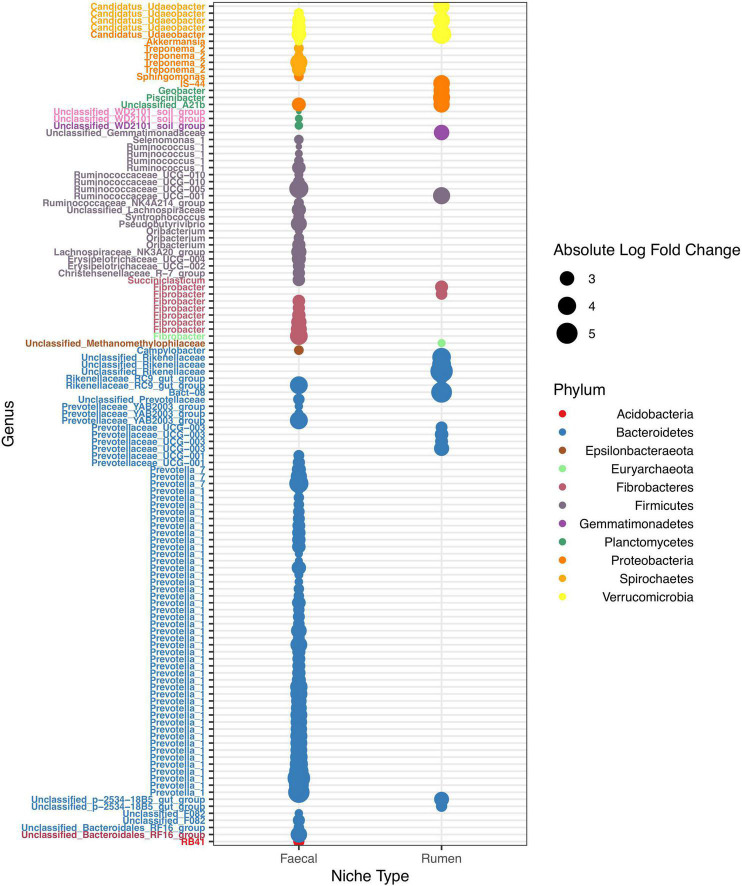
Dot plot of N-responsive ASV across niches. 16S ASVs with more than twofold change in abundance responsive to nitrogen treatments. Each differentially abundant ASV is shown as a dot coloured by phylum. Dot sizes illustrate the absolute fold change. The *X*-axis represents the niche type, namely faecal and rumen. The *Y*-axis represents the taxonomy classification of individual N-responsive ASV.

To identify changes in ASV abundance in response to nitrogen treatment, an Exact test was performed for each niche between two nitrogen treatment pairs: no-nitrogen verses medium-nitrogen, or no-nitrogen vs. high-nitrogen. For each niche, responsive ASVs (Exact test with logFC ≥ 2 or logFC ≤ −2, and BH adjusted *p* < 0.05) (defined as N-responsive ASVs) were identified. Scattered plots were used to illustrate abundance changes of N-responsive ASV across niches (Figure 3 and Supplementary Figures 5, 6). Volcano plots (Supplementary Figures 7–9) and heatmaps (Supplementary Figures 10–12) were used to provide complementary details on fold changes of N-responsive ASVs across niches and nitrogen treatments.

All N-responsive ASVs were linked with animal-associated niches (Figure 3 and Supplementary Figures 5, 6), but their presences were detected in other niches (Supplementary Figures 10–12). In 16S communities for example, 126 ASV were originally identified as N-responsive in animal-associated niches (Supplementary Table 4A and Figure 3), but none in other niches. Interestingly, over one third (47 out of 124) of N-responsive 16S ASVs were classified under the genus *Prevotella*. Moreover, all of the N-responsive *Prevotella* ASVs had positive foldchanges under medium- or high-nitrogen (Supplementary Table 4A). Similarly in 18S and ITS communities, the majority of N-responsive ASVs of (18S: 1692 out of 1694, ITS: 139 out of 140) were only responsive in animal-associated niches (Supplementary Figures 8, 9 and Supplementary Tables 4B,C). Surprisingly, no ASV from BS niche was N-responsive across amplicons. In addition, close to a third of 18S N-responsive ASVs (495 out of 1694) were unclassified, suggesting a lack of reference sequences for N-responsive 18S sequences.

### Nitrogen Treatment Drastically Reduced Microbial Network Connectivity in Soil but the Knock-On Effects on Other Niches Were Random

To investigate microbiome network changes in response to nitrogen treatment across niches, network analyses were performed for each niche individually with a pool of unique ASVs. The ASV pool was formed by a conjunction of N-responsive ASVs and the 200 most abundant ASVs (Supplementary Table 5) from each niche. Pairwise Spearman correlations were conducted between selected ASVs (i.e., ASVs found in ASV pool) to calculate their correlations. Only strong correlations (*r* ≥ 0.8 or *r*≤ −0.8, *p* ≤ 0.05, defined as connections) were included in the networks (Supplementary Table 6). Networks in each niche were formed by all connections found with each nitrogen treatment, each network connection was formed by two ASVs (i.e., nodes) and a line (i.e., edge) in between. Overall, microbiome networks were responsive to nitrogen treatment across niches, but responses were inconsistent between niches and across amplicons.

**FIGURE 4:**
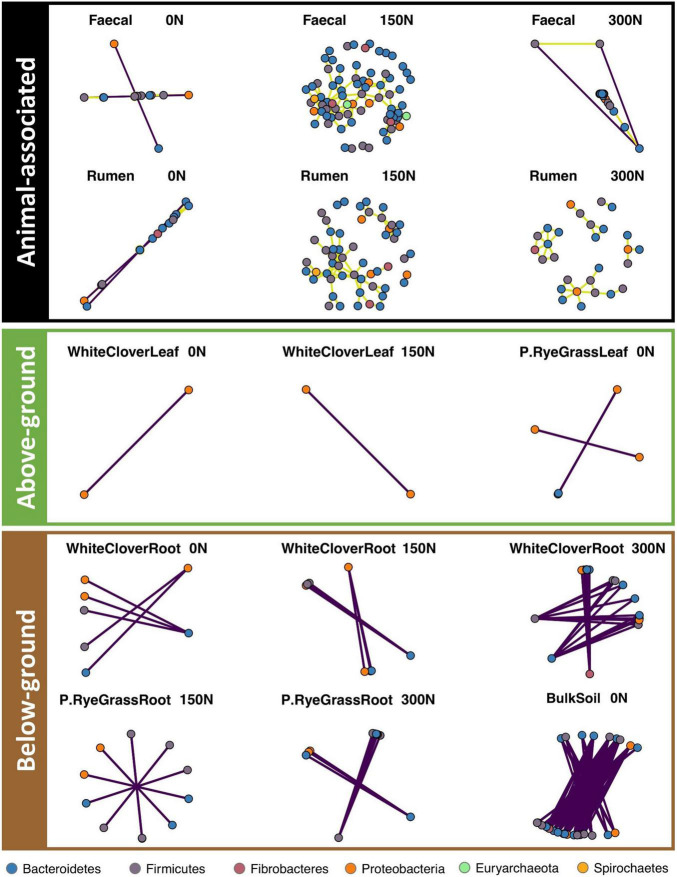
Microbiome networks (16S) across niche and N treatments. Pair-wise Spearman correlation is calculated for each ASV pair. Only strong significant correlations with *r* > 0.8 were kept and separated to present animal-associated (black), above-ground (green) and below-ground (brown) niches. Each dot represent an ASV, coloured by phylum. Purple edges represent negative correlation, olive yellow edges represent positive correlation.

Nitrogen treatment drastically reduced or eliminated network connections in BS across amplicons (Figure 4, Supplementary Figures 13–14, and Supplementary Table 7). On average, BS networks had 4.94, 9.43, and 16.21 edges per node in 16S, 18S, and ITS, respectively, under no-nitrogen. BS network connections were drastically reduced for ITS under medium- and high-nitrogen (2.86 and 3.03 edges per node, respectively), and were eradicated for 16S and 18S under medium- and high-nitrogen.

Nitrogen treatment had knock-on effects on other microbiome networks, but resulted in random changes across niches and amplicons (Figure 4 and Supplementary Figures 13, 14). For 16S, faecal networks under medium-nitrogen (108 nodes and 149 edges) were the most dense compared to networks under no- or high-nitrogen (no-nitrogen: 22 nodes and 20 edges; high-nitrogen: 76 nodes and 59 edges, Figure 4 and Supplementary Table 7). Similar trends were noted in the other 16S animal-associated networks, but not for other amplicons (Supplementary Figures 13, 14 and Supplementary Table 7). Rhizosphere networks responded to nitrogen treatment differently between niches and amplicons. Network connections of WCR in both 16S (18 nodes and 24 edges) and ITS (94 nodes and 700 edges) under high-nitrogen were the most dense compared to that under no- or medium-nitrogen (Figure 4, Supplementary Figure 14, and Supplementary Table 7). In contrast, RGR networks only showed notable changes in 18S, where networks (39 nodes and 55 edges) under medium-nitrogen were the most dense (Supplementary Figure 13). Phyllosphere microbiome networks across amplicons showed no response to nitrogen treatment.

## Discussion

Despite the growing interest of agricultural microbiomes and their responses to different agricultural management systems, the focus has been usually within specific niches, such as soil (Andrew et al., 2012; Mendes et al., 2015), rumen (Noel et al., 2019; Welty et al., 2019), or rhizosphere (Zhu et al., 2016; Huang et al., 2019). However, microbial interactions also play a critical role in many ecosystem process, especially nutrient cycling (Allison and Martiny, 2008), that may have carry over effects into other niches. Focusing only on the managed niche limits the ability to understand the consequences of management decisions and disturbances to other nearby niches. Therefore, the current study focused on microbiomes of soil under nitrogen treatment, as well as microbiomes in the nearby niches, to investigate potential relationships between niche microbiomes.

### Nitrogen Fertilisation Had No Impact on Microbial Diversity and Composition

Microbiome diversity and community composition had no response to nitrogen treatment in the soil niche itself or in any other niche within the same farm. This is consistent with previous studies (Eo and Park, 2016; Banerjee et al., 2019; Zhao et al., 2019), but not with many others (Ramirez et al., 2012; Zeng et al., 2016; Wang et al., 2018). Zhou et al. (2016) suggested that long term nitrogen fertilisation significantly decrease fungal diversity and alter fungal compositions in soil, but our results suggested much weaker effects. The disagreement could be caused by the heterogeneity of soil microbiomes between different research sites. Unlike rumen or gut microbiomes, which were relatively more defined, soil microbiomes varied both spatially and temporally (Ettema and Wardle, 2002; Martiny et al., 2011; Pasternak et al., 2013). Besides, certain organisms could be more sensitive to nitrogen treatments than others. Zhao et al. (2019) suggested that the overall fungal community composition and diversity were not affected by nitrogen treatments, but around 10% of taxa were sensitive to nitrogen treatment. Furthermore, only a small number of soil samples were taken in the current study. This potentially reduced the statistical power to detect significant distinctions between microbiomes under different nitrogen treatments.

Plant associated (phyllosphere and rhizosphere) microbial community compositions and diversities were not responsive to nitrogen treatments except 16S microbiomes in WCR. Ledgard et al. (2001) suggested that nitrogen additions (400 kg/ha/yr) reduced the productivity of nitrogen fixation in white clovers. The symbiosis between white clover root and *Rhizobium* are fundamental for nitrogen fixation (Kneip et al., 2007), and nitrogen fertilisation can make this capability redundant, thus leading to compositional changes in root communities, such as a lower proportion of nitrogen fixers.

Our results showed that soil nitrogen treatment did not affect microbiome richness and compositions in animal-associated niches. It was unlikely that animal-associated microbiomes were experiencing direct changes of nitrogen availability. In contrast, the soil nitrogen treatment would most likely influence ruminants by changing the plant materials grazed on the pasture (Henderson et al., 2015). Applications of nitrogen fertiliser was proven to favour pasture growth while the production of clover was unfavourable (Moir et al., 2003), leading to a higher ryegrass: white clover ratio. This vegetation change would then lead to changes in nutrient intake to a higher fibre and less protein diet for ruminants (Rattray and Joyce, 1974). Bowen et al. (2018) and Smith et al. (2020) showed that changes in sward types (i.e., ryegrass only vs. combined ryegrass and white clover) altered the rumen microbiome composition in dairy cows. In contrast, O’Callaghan et al. (2018) suggested that changes in feedings between ryegrass only and mixture of ryegrass and white clover had no impact on rumen microbiome composition, but altered the rumen metabolome. Unlike the previous microbiome studies on sward type alterations (i.e., with or without white clover) (Bowen et al., 2018; Smith et al., 2020), we did not expect to see a huge change in ryegrass and white clover ratio between control and fertilised ground [i.e., about 5–10% reduction in white clover on fertilised ground (Moir et al., 2003)]. This small change in vegetation might not lead to a detectable change in animal microbiomes.

### N-Responsive Amplicon Sequence Variants Were Observed in Animal Associated Niches, but Not in Other Niches

Many N-responsive ASVs were present across niches (Supplementary Figures 10–12), but most of N-responsive ASVs only had strong correlations in animal-associated niches across amplicons, and few N-responsive ASVs in phyllosphere or rhizosphere niches (Supplementary Figures 5, 6). Zancarini et al. (2012) suggested that soil nitrogen treatments only affected rhizosphere microbiomes with certain host plant genotypes (Zancarini et al., 2012). Therefore, our results could mean that white clover and rye grass rhizosphere microbiomes were insensitive to soil nitrogen treatments. Beattie and Lindow (1999) stated that phyllosphere microorganisms were generally oligotrophs which can tolerate low-nutrient conditions or formed symbiosis with host plant to obtain more nutrients. When obtaining nutrient from soil, physical movement of nutrients, most likely from host roots to leaf surface, was inevitable for phyllosphere microbiomes. Hence, phyllosphere microbiomes might not experience much knock-on effects caused by soil nitrogen treatments due to physical barriers.

Among 16S N-responsive ASVs, over one-third (47 out of 126) were classified as *Prevotella* spp., all of which were enriched under medium- or high-nitrogen compared to no-nitrogen, suggesting that *Prevotella* spp. were potentially benefited from soil nitrogen treatment, or vegetation change (i.e., 5 – 10% reduction of white clover). Compared to white clover, ryegrass contained less protein and more soluble and structural carbohydrates (Rattray and Joyce, 1974). *Prevotella* was known to be an abundant and essential group of catabolic generalists in the rumen (Hungate, 2013; Emerson and Weimer, 2017), due to the ability of degrading a variety of carbohydrate substrates, including hemicellulose, xylan, and pectin (Dodd, 2010). Therefore, the enrichment of N-responsive *Prevotella* spp. was most likely due to increases in carbohydrates, or in other words higher ryegrass intake by cows. Unlike *Prevotella* spp., patterns were random for other N-responsive taxa. This suggested that the organisms under the same taxa groups could respond to the same disturbance differently. For example, *Trichostomatia* is a common group of rumen protozoa fermenters involved in degrading non-structural polysaccharides and soluble sugar (Wright, 2015). This aligned with our results as 696 out of 1199 N-responsive ASVs were *Trichostomatia* ASVs. Furthermore, because the range of carbohydrates metabolised was genus-dependent (Wright, 2015), it was unsurprising to see inconsistent change in N-responsive *Trichostomatia* ASVs.

### Microbiome Networks Were Responsive to Nitrogen Treatments but With No Consistent Pattern

Our results showed that for 16S and 18S communities, network connections in soil were completely eradicated under medium- and high-nitrogen, whereas ITS networks complexity and connectivity were drastically reduced but still detectable. Our findings aligned with a previous study showing agricultural fertilisation associated with decreases in microbial network complexity (Banerjee et al., 2019). The dramatic reduction in network linkages in response to nitrogen could be due to the redundancy of nitrogen-fixation functions. When nitrogen was limited in soil, microorganisms formed networks to transform inaccessible nitrogen into an accessible form for non-nitrogen fixers, supporting community growth (Kuypers et al., 2018). Under medium- or high-nitrogen, potentially non-nitrogen fixers would no longer require nitrogen fixation from nearby nitrogen fixers, leading to an alteration of microbiome networks. Instead, alternative nitrification pathways, such as aerobic ammonia oxidation, could potentially be induced and consequently led to population and activity increases of ammonia-oxidizing microbes (Di et al., 2009; Dai et al., 2013). However, further investigations on the transcriptome are required to corroborate the conjecture.

Other than reduction of soil network connectivity, ITS community networks were unresponsive to nitrogen treatment across niches, suggesting that fungal communities were less sensitive to nitrogen treatments compared to prokaryotes and other eukaryotes in general. However, since the current study was only based on amplicon sequencing, the presence of ‘relic fungal DNA’, such as dormant fungal spores, was potentially interfere with detection of relevant fungi and confound the results.

## Conclusion

Our results showed that urea-based nitrogen fertilisation of soils did not affect microbiome diversity or composition in soil and neighbouring niches, but does affect microbiome networks and potential interactions. Soil microbiome networks from all three amplicons were drastically reduced under medium- and high-nitrogen. Microbiome networks across neighbouring niches, such as rumen and faecal, were also responsive to nitrogen treatments despite no direct management. However, changes of microbiome networks were varied between niches, suggesting differentiations in reconstruction mechanisms between microbiomes across niches. The present study showed that treating one single niche in a farm could lead to knock-on effects on neighbouring microbiomes. This study would be of value to further evaluate the effects of treatments or disturbances in agricultural land and potentially in other various ecosystems.

## Limitations

One limitation of the current study was the low statistical power in non-animal niches due to a small sample size. Other than rumen (*n* = 52) and faecal (*n* = 62) samples, no other niche had more than 12 samples (i.e., WhiteCloverLeaf: *n* = 12, WhiteCloverRoot: *n* = 11, P.RyeGrassLeaf : *n* = 9, P.RyeGrassRoot : *n* = 12, BulkSoil : *n* = 11), making the statistical tests less reliable. Another limitation was the potential existence of “relic DNA” in soil (Carini et al., 2016), especially for fungal communities. Depending on the organisms, Carini et al. (2016) suggested that “relic DNA” could persist in soil for weeks to years depending on the type of dead organisms. Unlike most prokaryotes or plants, fungal spores were known to be long-lived (Ziemer et al., 2002), and widely dispersed (Hallenberg and Kúffer, 2001). This complicated our results, as we could not be certain all the ITS sequences detected in the study were from live local fungal species. Therefore, further RNA work is necessary to resolve the potential confounding factor caused by potential “relic DNA.”

## Data Availability Statement

The datasets presented in this study can be found in online repositories. The names of the repository/repositories and accession number(s) can be found in the article/Supplementary Material.

## Ethics Statement

The animal study was reviewed and approved by Animal Welfare Act 1999 via Lincoln University. Written informed consent was obtained from the owners for the participation of their animals in this study.

## Author Contributions

GA, G-AG, PG, and SM conceived the study and participated in its design. KR, RH, LJ, G-AG, ST, PG, AP, AB, NP, and GA collected the samples and conducted the experiments. XW analysed the data, wrote, and illustrated the manuscript with input from all other authors. All authors contributed to the article and approved the submitted version.

## Conflict of Interest

KR, AB, LJ, ST, NP, and GA were employed by the company AgResearch Ltd. The remaining authors declare that the research was conducted in the absence of any commercial or financial relationships that could be construed as a potential conflict of interest.

## Publisher’s Note

All claims expressed in this article are solely those of the authors and do not necessarily represent those of their affiliated organizations, or those of the publisher, the editors and the reviewers. Any product that may be evaluated in this article, or claim that may be made by its manufacturer, is not guaranteed or endorsed by the publisher.

## Supplementary Material

Supplementary Figure 1 18S Microbiome diversities and composition comparisons across farm niches under three levels of nitrogen treatments (0, 150, and 300 N/ha/yr). **(A)** Microbiome richness based on number of observed ASVs are shaped by nitrogen treatments. Open circle , plus symbol +, and closed triangle▲ represent 0, 150, and 300 N/ha/yr nitrogen managements, respectively. Statistical significances shown in figure were calculated with Kruskal-Wallis test. **(B)** Relative abundance of microbiome taxa across farm niches coloured at Phylum level. **(C)** NMDS plot using Bray-Curtis distance coloured by niches and shaped by treatment levels (ANOSIM: *p* < 0.01, *R* = 0.246 and ADONIS: *p* < 0.01, *R*^2^ = 0.248).

Supplementary Figure 2 ITS Microbiome diversities and composition comparisons across farm niches under three levels of nitrogen treatments (0, 150, and 300 N/ha/yr). **(A)** Microbiome richness based on number of observed ASVs are shaped by nitrogen treatments. Open circle , plus symbol +, and closed triangle▲ represent 0, 150, and 300 N/ha/yr nitrogen managements, respectively. Statistical significances shown in figure were calculated with Kruskal-Wallis test. **(B)** Relative abundance of microbiome taxa across farm niches coloured at Phylum level. **(C)** NMDS plot using Bray-Curtis distance coloured by niches and shaped by treatment levels (ANOSIM: *p* < 0.01, *R* = 0.647, ADONIS: *p* < 0.01, *R*^2^ = 0.325).

Supplementary Figure 3 Microbiome frequency and occupancy plot (18S), illustrating the different number of populations occupying different number of samples at each niche. *X*-axis illustrates number of sample sites panelled by each individual niche. *Y*-axis illustrates accumulated number of present ASV in certain number of samples.

Supplementary Figure 4 Microbiome frequency and occupancy plot (ITS), illustrating the different number of populations occupying different number of samples at each niche. *X*-axis illustrates number of sample sites panelled by each individual niche. *Y*-axis illustrates accumulated number of present ASV in certain number of samples.

Supplementary Figure 5 Dot plot of N-responsive ASV across niches. 18S ASVs with more than eightfold change in abundance responsive to nitrogen treatments. Each differentially abundant ASV is shown as a dot coloured by phylum. Dot sizes illustrate the absolute fold change. The *X*-axis represents the niche type, namely faecal and rumen. The *Y*-axis represents the taxonomy classification of individual N-responsive ASV.

Supplementary Figure 6 Dot plot of N-responsive ASV across niches. ITS ASVs with more than twofold change in abundance responsive to nitrogen treatments. Each differentially abundant ASV is shown as a dot coloured by phylum. Dot sizes illustrate the absolute fold change. The *X*-axis represents the niche type, namely faecal and rumen. The *Y*-axis represents the taxonomy classification of individual N-responsive ASV.

Supplementary Figure 7 Volcano plot (16S) showing details on ASVs with more than twofold change in abundance responsive to nitrogen treatments across niches and N treatments. Each N-responsive ASV is shown as a dot coloured by phylum. Positive or negative responses in log fold change for each N-responsive ASV is illustrated by the *X*-axis. The *Y*-axis represents the -log10 adjusted *p*-value (*p* < −0.05 across all ASVs showed in the figure).

Supplementary Figure 8 Volcano plot (18S) showing details on ASVs with more than twofold change in abundance responsive to nitrogen treatments across niches and N treatments. Each N-responsive ASV is shown as a dot coloured by phylum. Positive or negative responses in log fold change for each N-responsive ASV is illustrated by the *X*-axis. The *Y*-axis represents the −log10 adjusted *p*-value (*p* < −0.05 across all ASVs showed in the figure). NA means the ASV is N-responsive but unclassified at phylum level.

Supplementary Figure 9 Volcano plot (ITS) showing details on ASVs with more than twofold change in abundance responsive to nitrogen treatments across niches and N treatments. Each N-responsive ASV is shown as a dot coloured by phylum. Positive or negative responses in log fold change for each N-responsive ASV is illustrated by the *X*-axis. The *Y*-axis represents the −log10 adjusted *p*-value (*p* < −0.05 for all ASVs in the figure). NA means the ASV is N-responsive but unclassified at phylum level.

Supplementary Figure 10 Presence-absence heatmap (16S) showing the existence of N-responsive ASVs across multiple niches. The *X*-axis represents the N-treatment for all samples (there are multiple samples under the same treatment). The *Y*-axis represents the taxonomy classification of individual N-responsive ASV.

Supplementary Figure 11 Presence-absence heatmap (18S) showing the existence of N-responsive ASVs across multiple niches. The *X*-axis represents the N-treatment for all samples (there are multiple samples under the same treatment). The *Y*-axis represents the taxonomy classification of individual N-responsive ASV.

Supplementary Figure 12 Presence-absence heatmap (ITS) showing the existence of N-responsive ASVs across multiple niches. The *X*-axis represents the N-treatment for all samples (there are multiple samples under the same treatment). The *Y*-axis represents the taxonomy classification of individual N-responsive ASV.

Supplementary Figure 13 Microbiome networks (18S) across niche and N treatments. Pair-wise Spearman correlation is calculated for each ASV pair. Only strong significant correlations with *r* > 0.8 were kept. Each dot represent an ASV, coloured by phylum. Purple edges represent negative correlation, olive yellow edges represent positive correlation.

Supplementary Figure 14 Microbiome networks (ITS) across niche and N treatments. Pair-wise Spearman correlation is calculated for each ASV pair. Only strong significant correlations with *r* > 0.8 were kept. Each dot represent an ASV, coloured by phylum. Purple edges represent negative correlation, olive yellow edges represent positive correlation.

Supplementary Table 1 Sample metadata table.

Supplementary Table 2 For each amplicon type (16S, 18S, and ITS microbiomes), statistical summary on number of observed ASVs for each niche type across treatments. The mean, median and standard deviation values are rounded to one decimal place.

Supplementary Table 3 Relative abundance table summarised at Phylum level. Minor phylum (relative abundance < 0.01) are not showing in the table. **(A)** Phylum relative abundance at for 16S. **(B)** Phylum relative abundance at for 18S. **(C)** Phylum relative abundance at for ITS.

Supplementary Table 4 Exact tests results for all N-responsive ASV. For each N-responsive ASV, taxonomy classification, Log fold change (LogFC), log counts per million (logCPM), *p*-value, adjusted *p*-value (FDR), data comparisons between which two nitrogen treatments, and niche type are showed in different columns. Adjusted *p*-values are used for determining significance of ASV responses to nitrogen treatments.

Supplementary Table 5 ASV table (absolute abundance) showing all chosen N-responsive ASVs for network analyses across niches. **(A)** Abundance table of chosen ASV for 16S. **(B)** Abundance table of chosen ASV for 18S. **(C)** Abundance table of chosen ASV for ITS.

Supplementary Table 6 Network analyses results on Spearman rank correlation coefficient test across niches and nitrogen treatments. Analyses results for each amplicon is displayed in individual spreadsheet (**A**: 16S, **B**: 18S, and **C**: ITS).

Supplementary Table 7 Summary of network details across amplicons, niches, and nitrogen treatments. Number of nodes and edges, average number of edges per nodes, and the standard deviations are provided in the table.

## Abbreviations

ASV: amplicon sequence variant
BS: bulk soil
DM: dry matter
F: faecal
ITS: internal transcribed spacer
N: nitrogen
R: rumen
RGL: ryegrass leaf
RGR: ryegrass root
RPM: rising-plate meter
rRNA: ribosomal RNA
WCL: white clover leaf
WCR: white clover root.
