# Supplementary material for: Soil Nitrogen Treatment Alters Microbiome Networks Across Farm Niches
Source: Front Microbiol. 2022 Feb 14;12:786156. doi: 10.3389/fmicb.2021.786156 (PMC8882991; doi:10.3389/fmicb.2021.786156)
Supplement: Supplementary Figure 1 — 18S Microbiome diversities and composition comparisons across farm niches under three levels of nitrogen treatments (0, 150, and 300 N/ha/yr). (A) Microbiome richness based on number of observed ASVs are shaped by nitrogen treatments. Open circle , plus symbol +, and closed triangle▲ represent 0, 150, and 300 N/ha/yr nitrogen managements, respectively. Statistical significances shown in figure were calculated with Kruskal-Wallis test. (B) Relative abundance of microbiome taxa across farm niches coloured at Phylum level. (C) NMDS plot using Bray-Curtis distance coloured by niches and shaped by treatment levels (ANOSIM: p < 0.01, R = 0.246 and ADONIS: p < 0.01, R2 = 0.248). [file Data_Sheet_1.pdf]

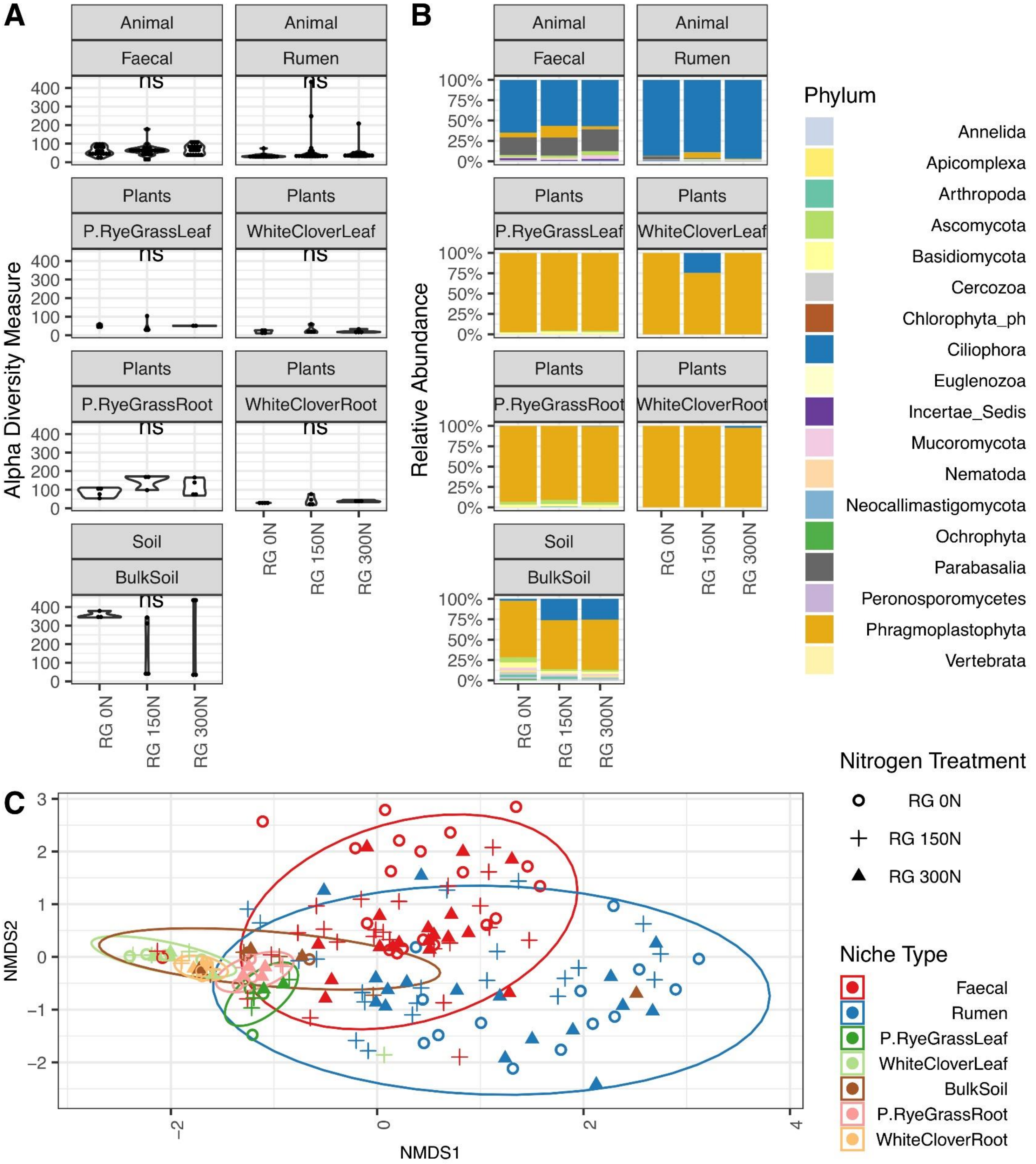

Supplementary Figure S1

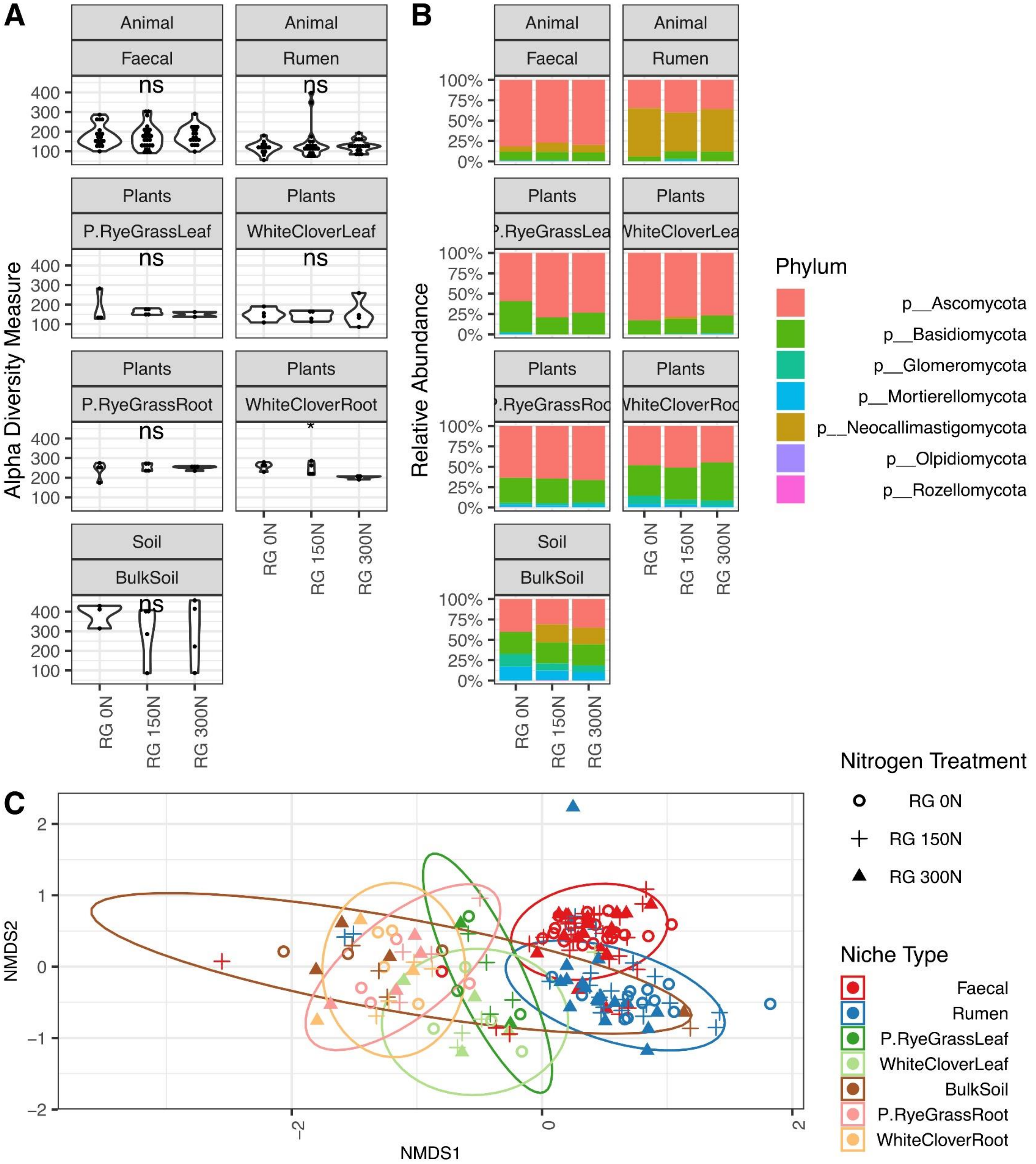

Supplementary Figure S2

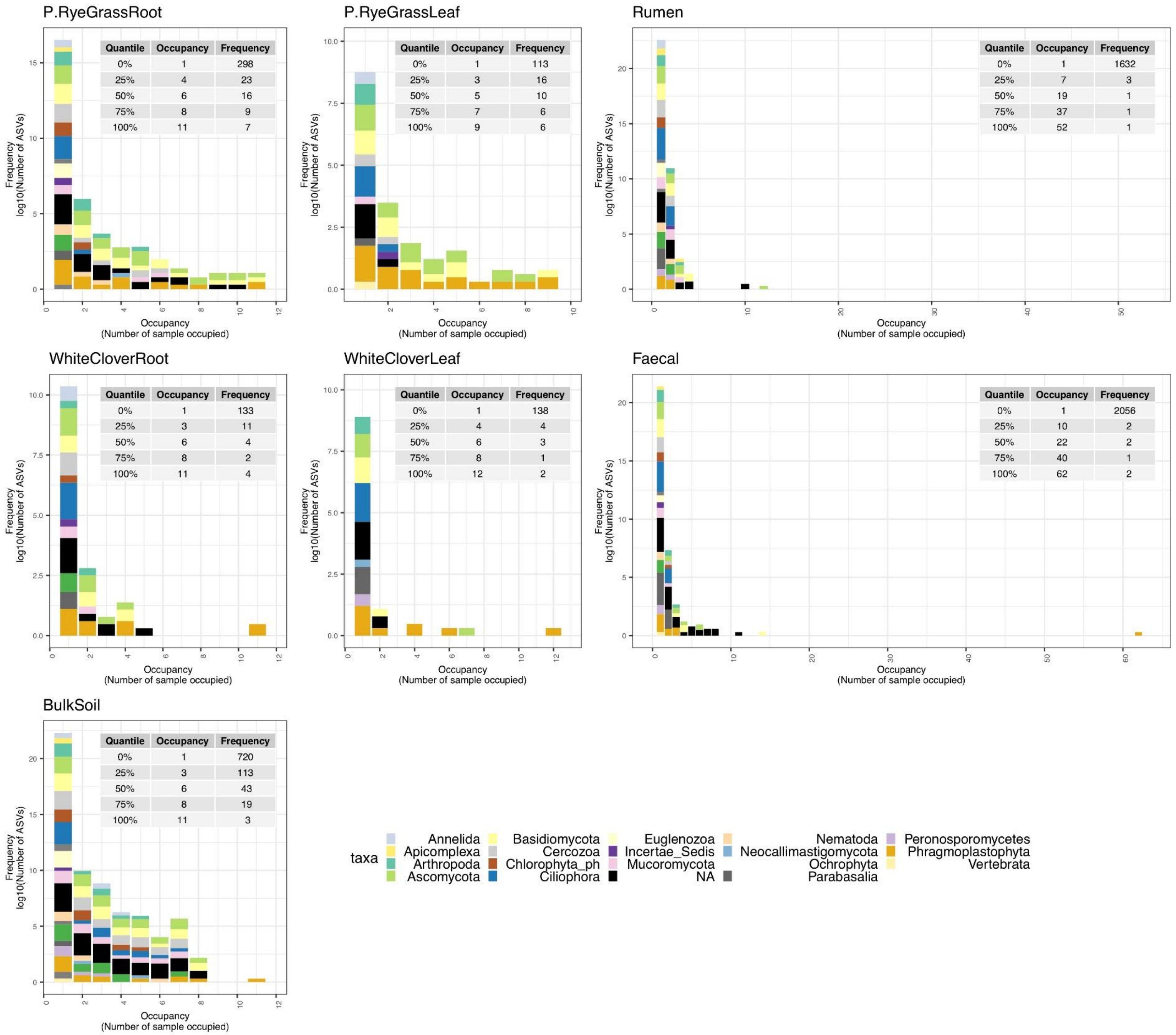

Supplementary Figure S3

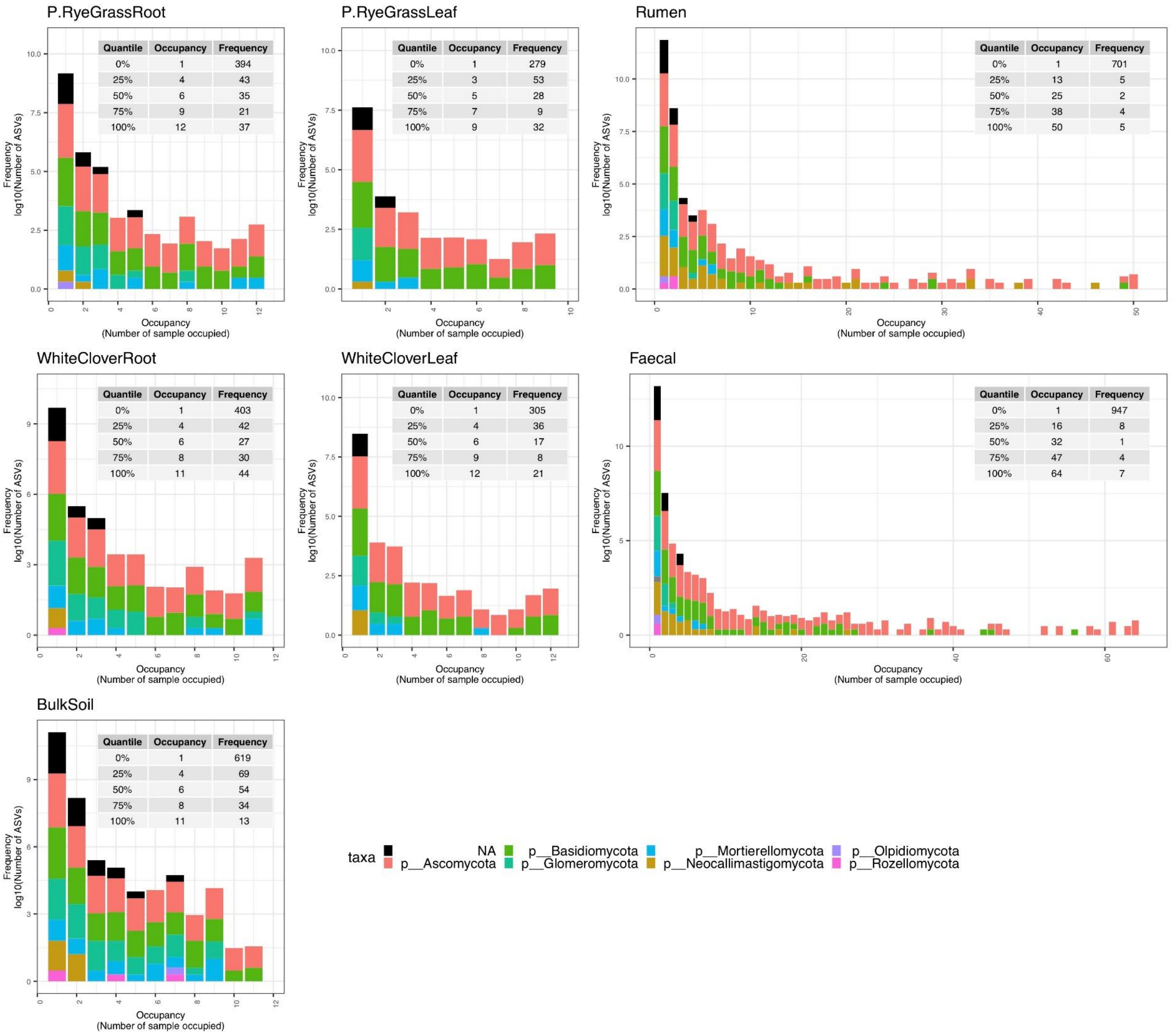

Supplementary Figure S4

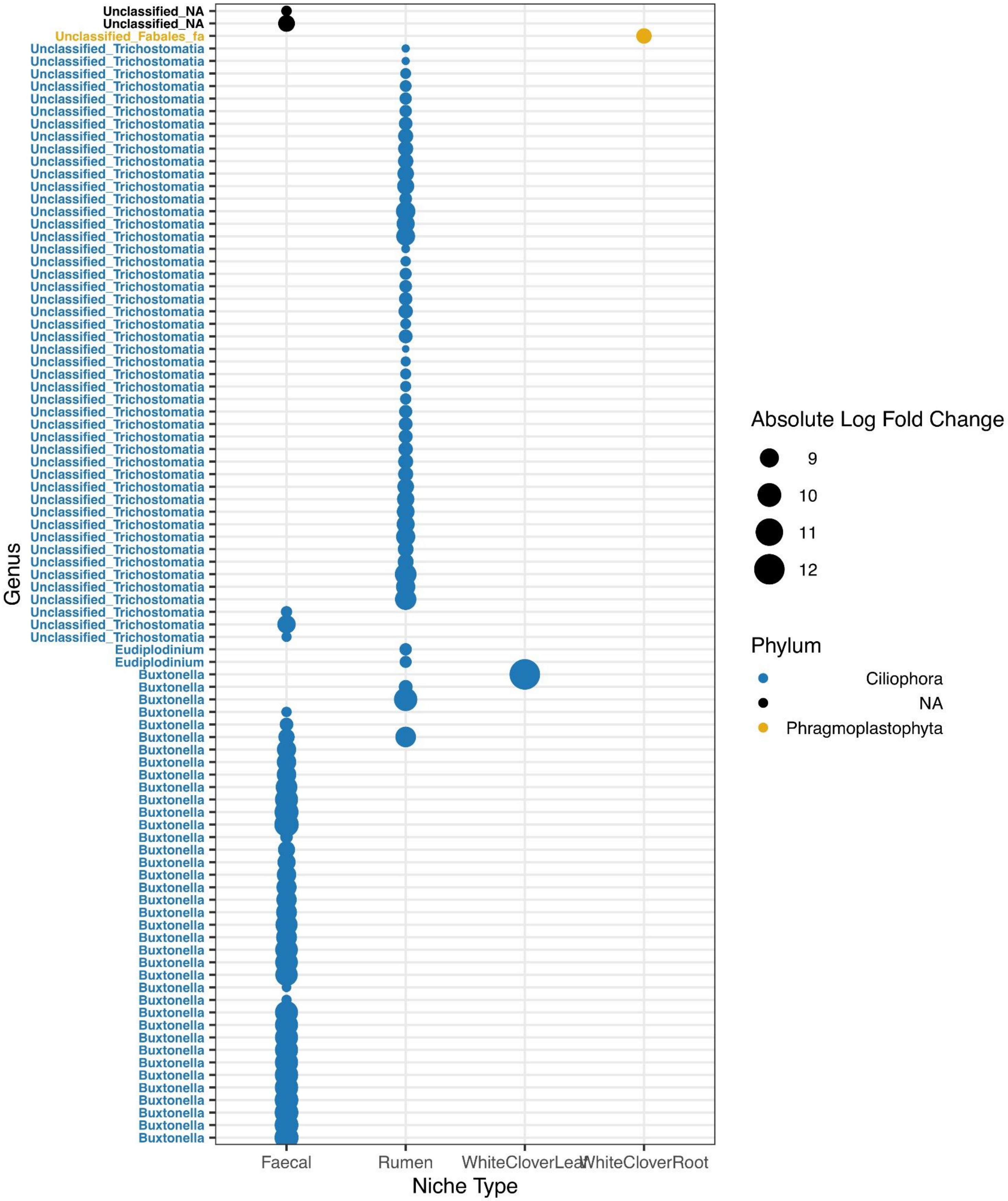

Supplementary Figure S5

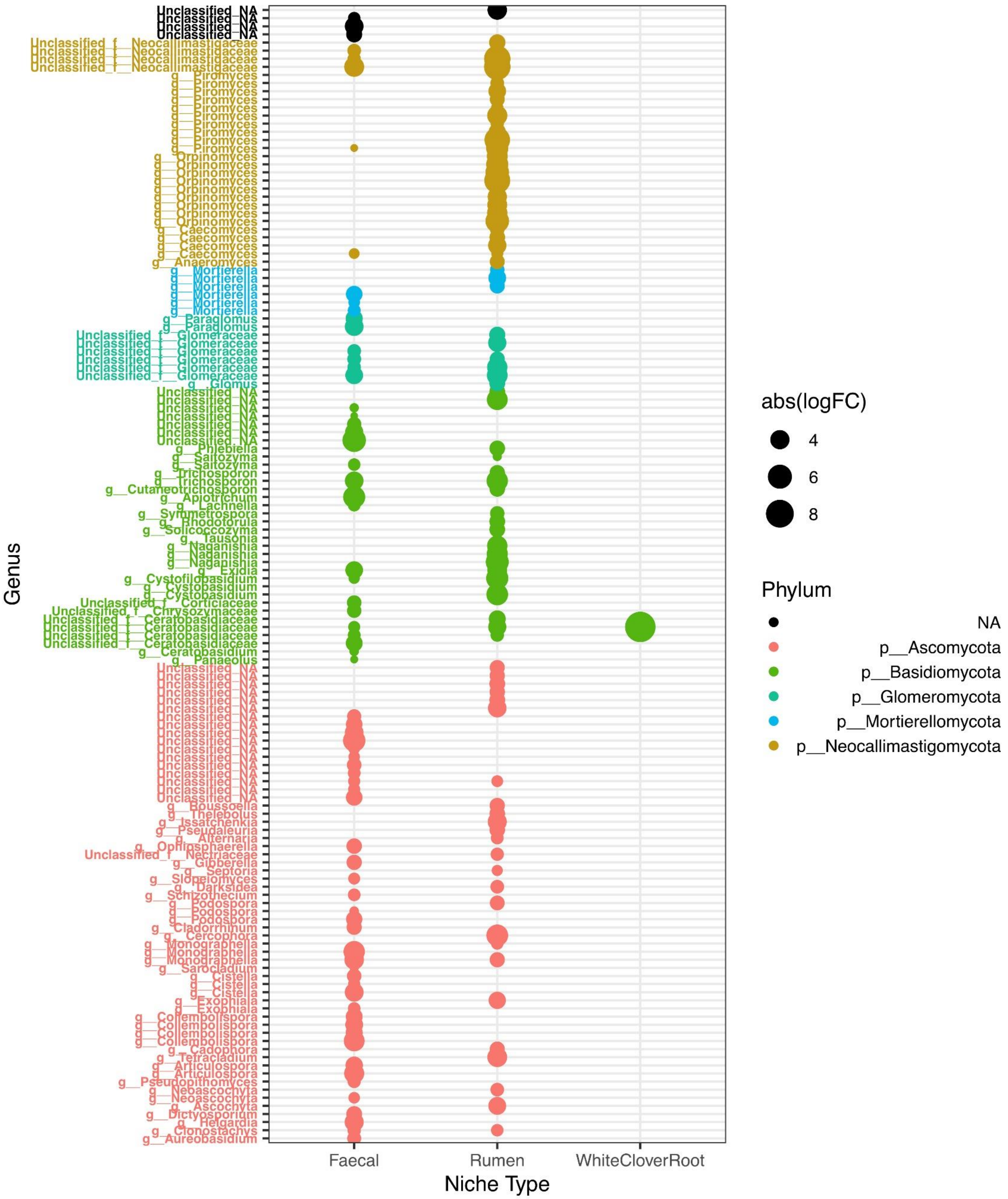

Supplementary Figure S6

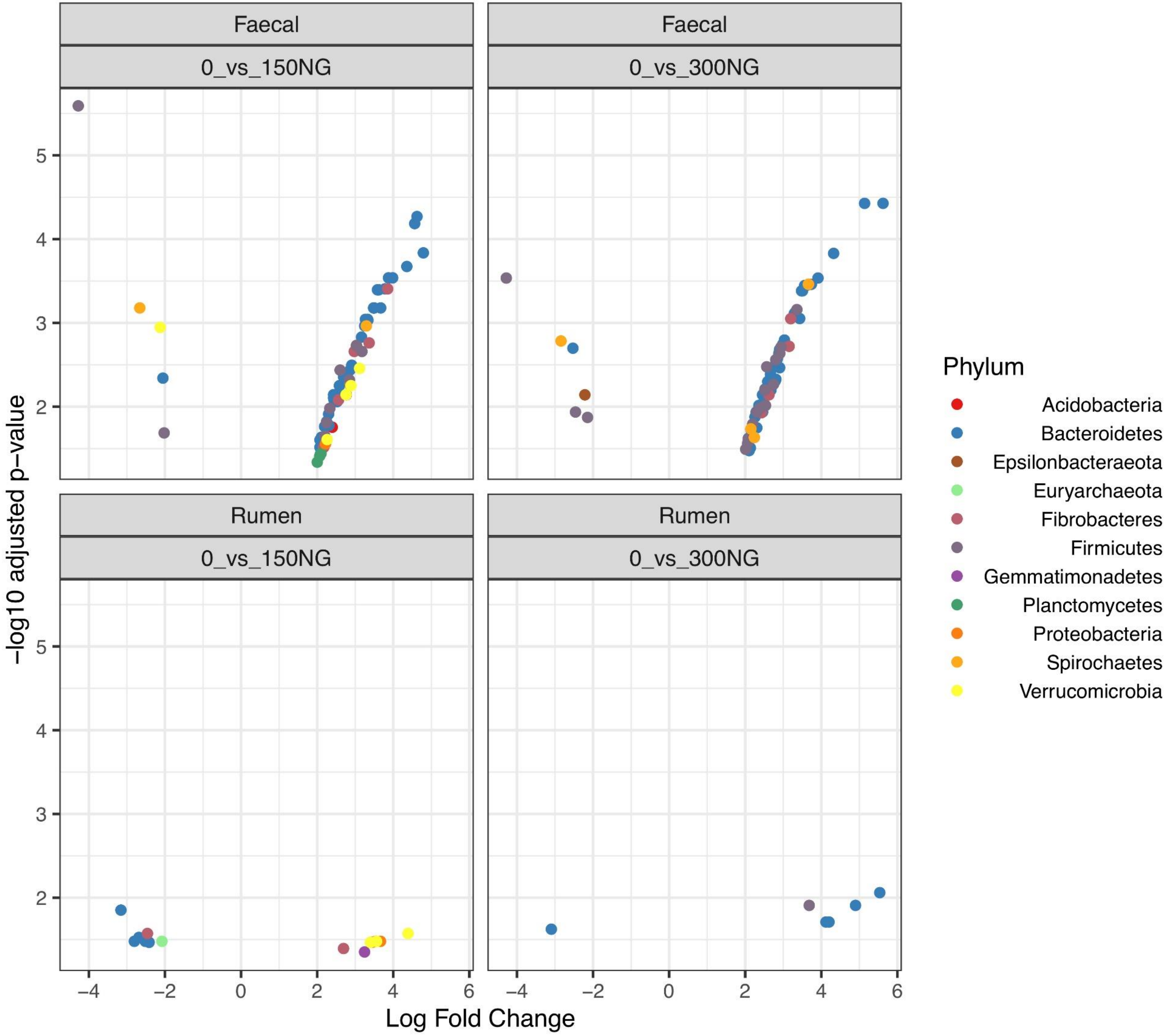

Supplementary Figure S7

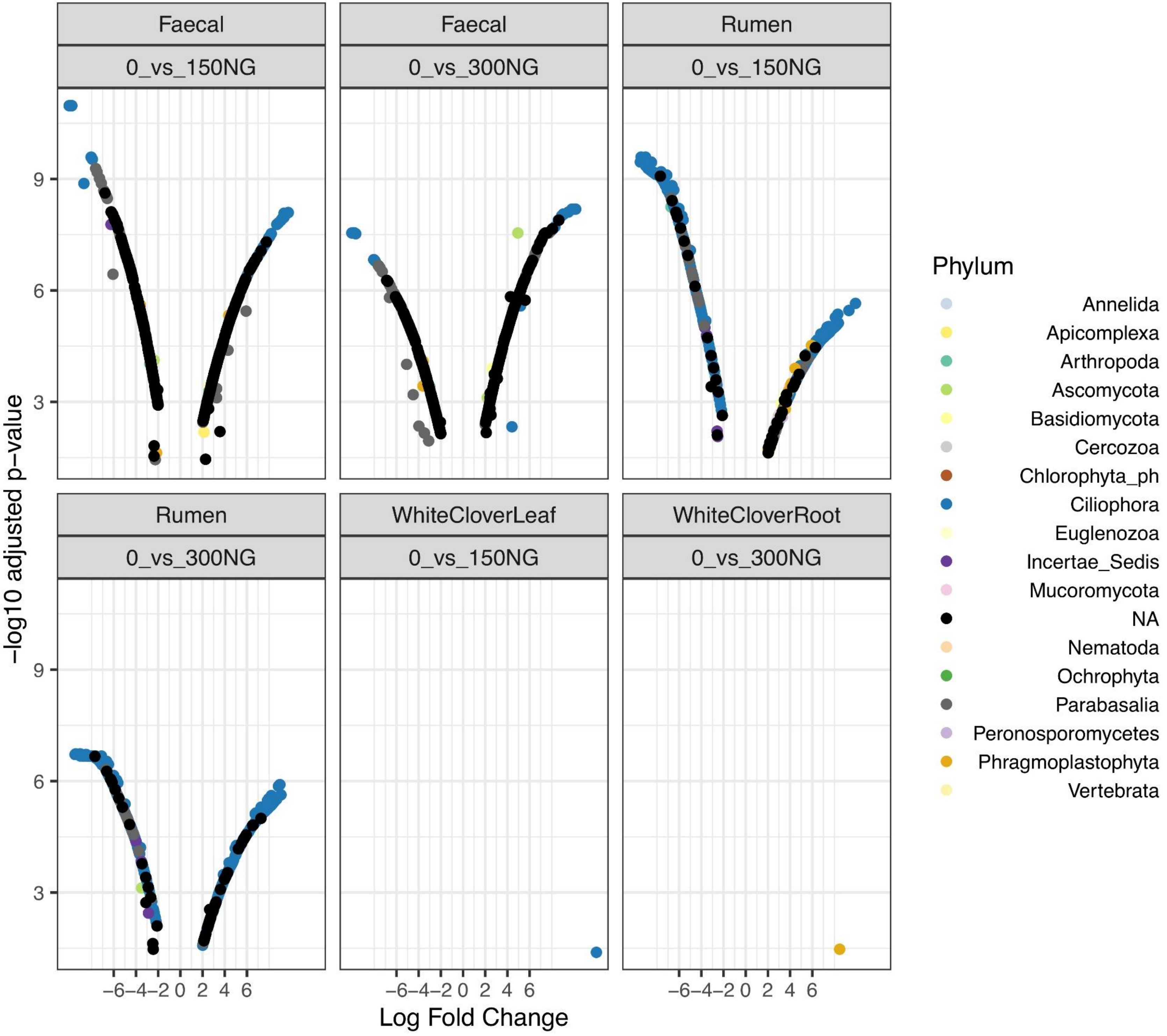

Supplementary Figure S8

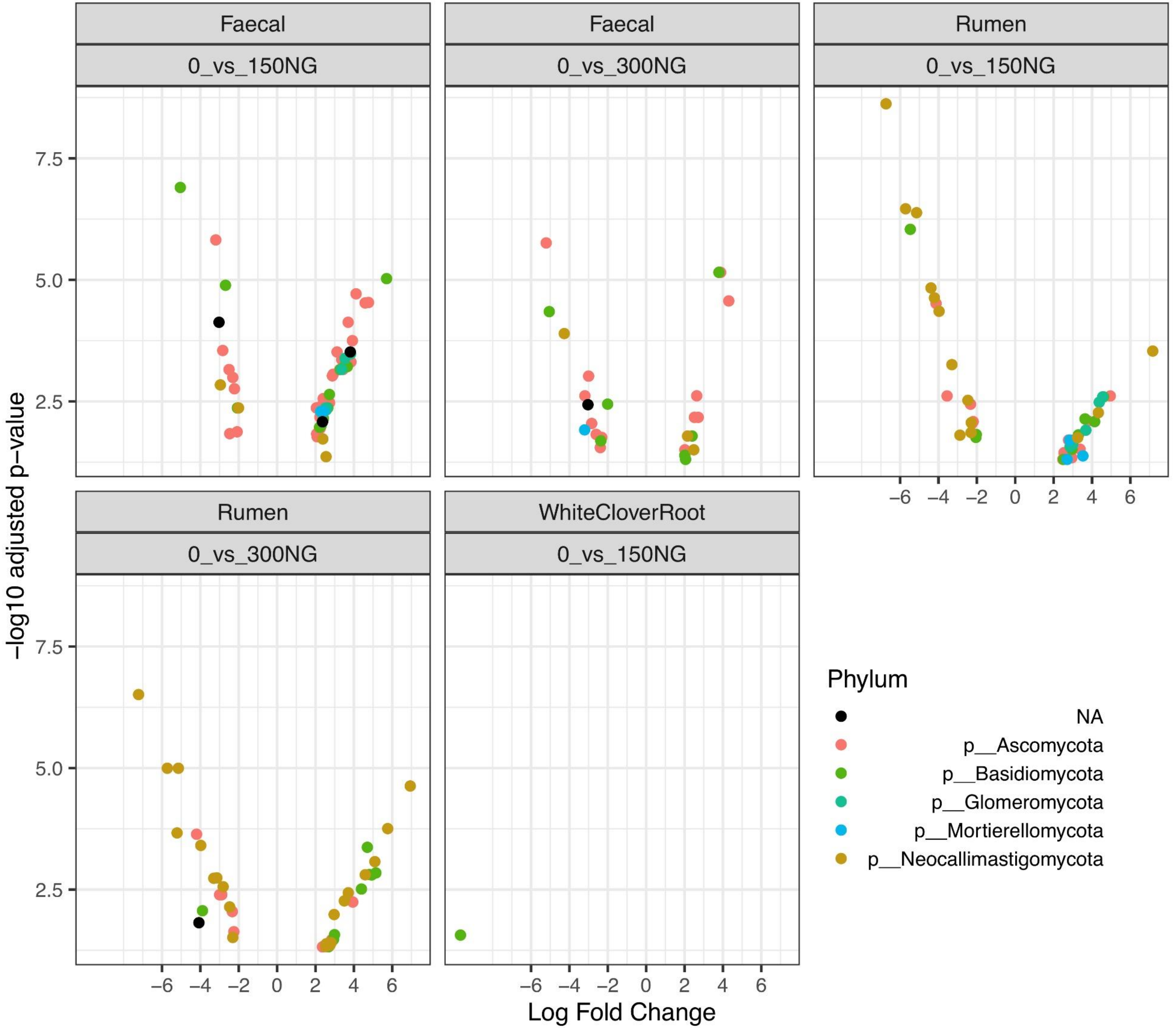

Supplementary Figure S9

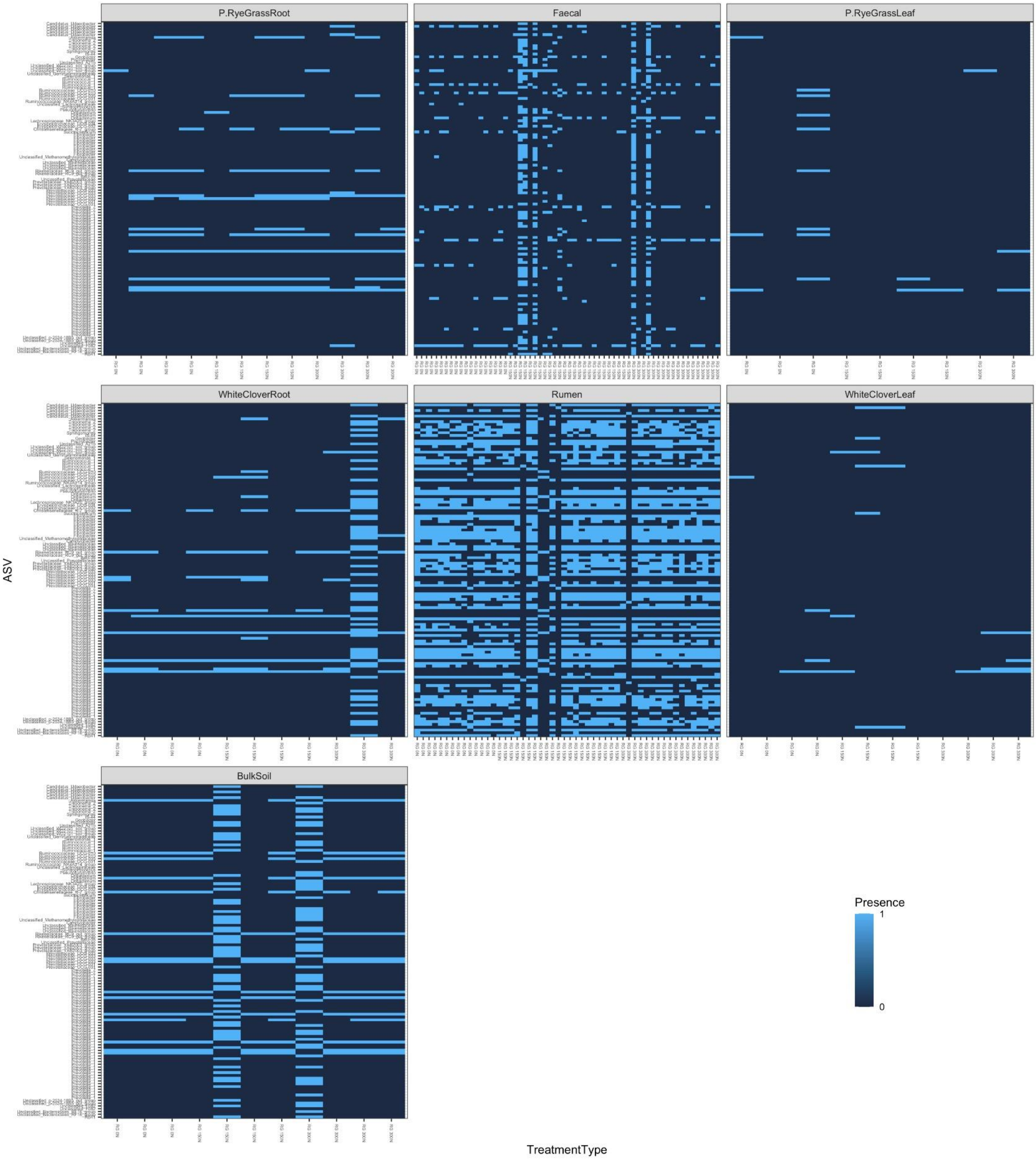

Supplementary Figure S10

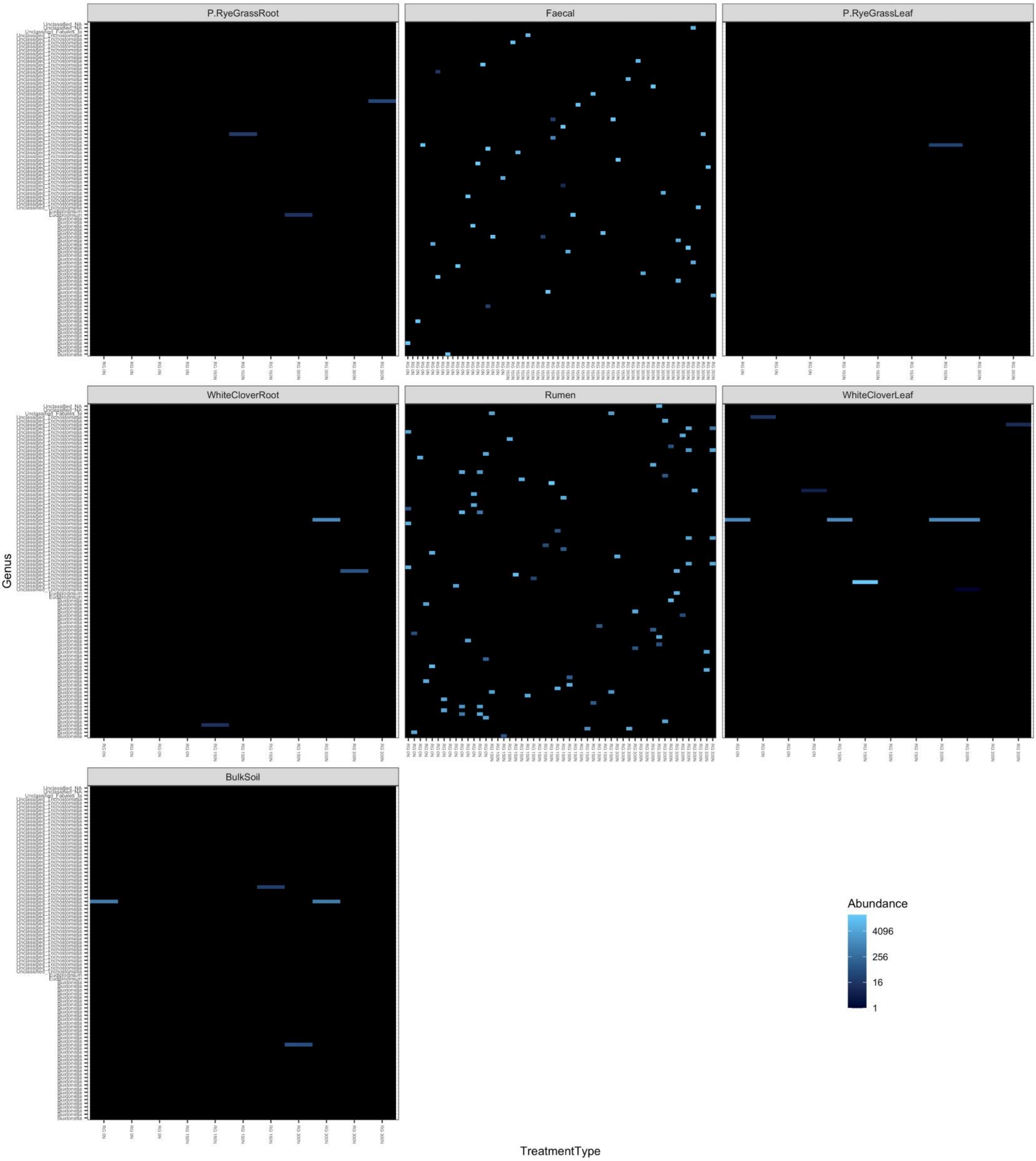

Supplementary Figure S11

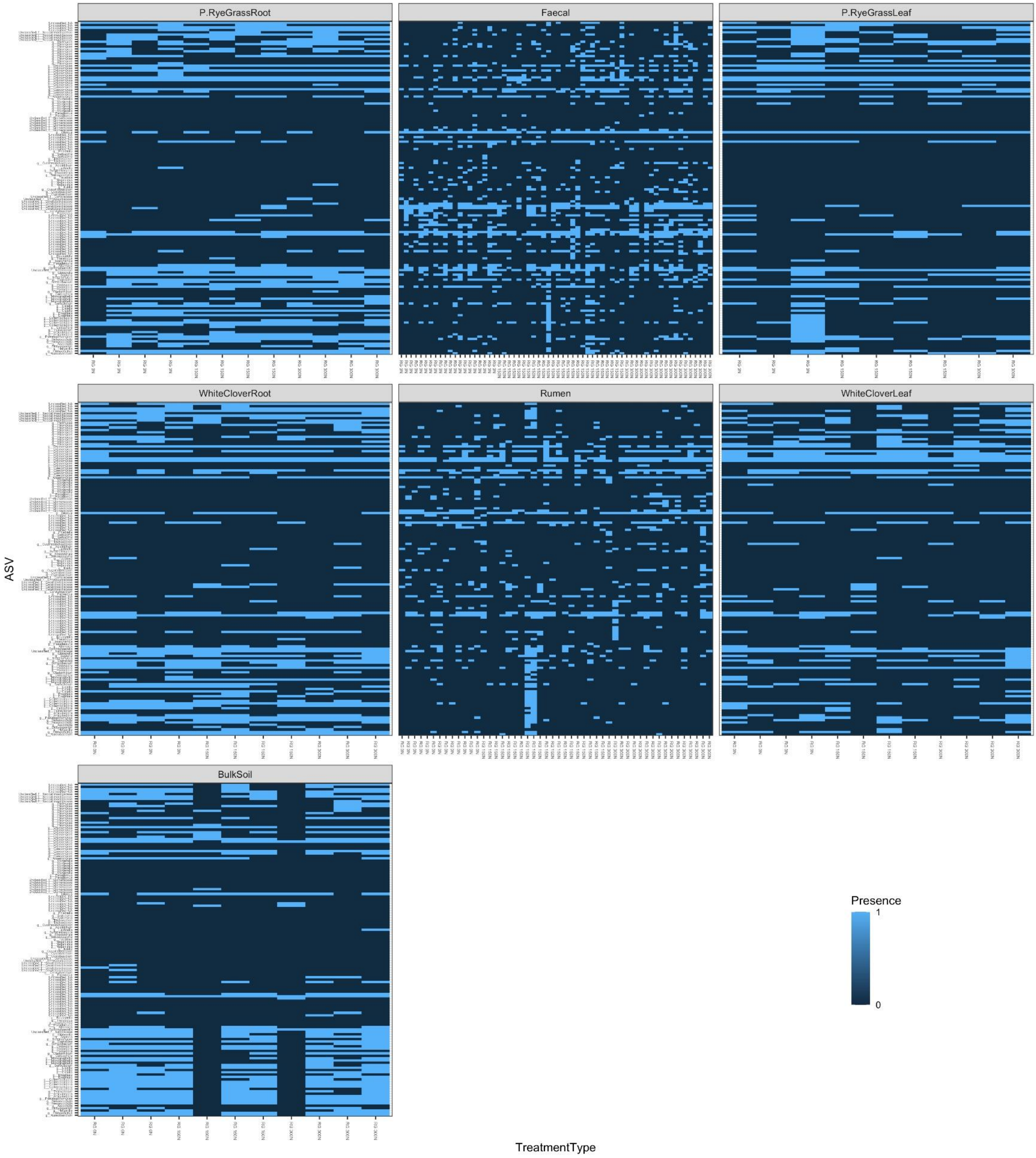

Supplementary Figure S12

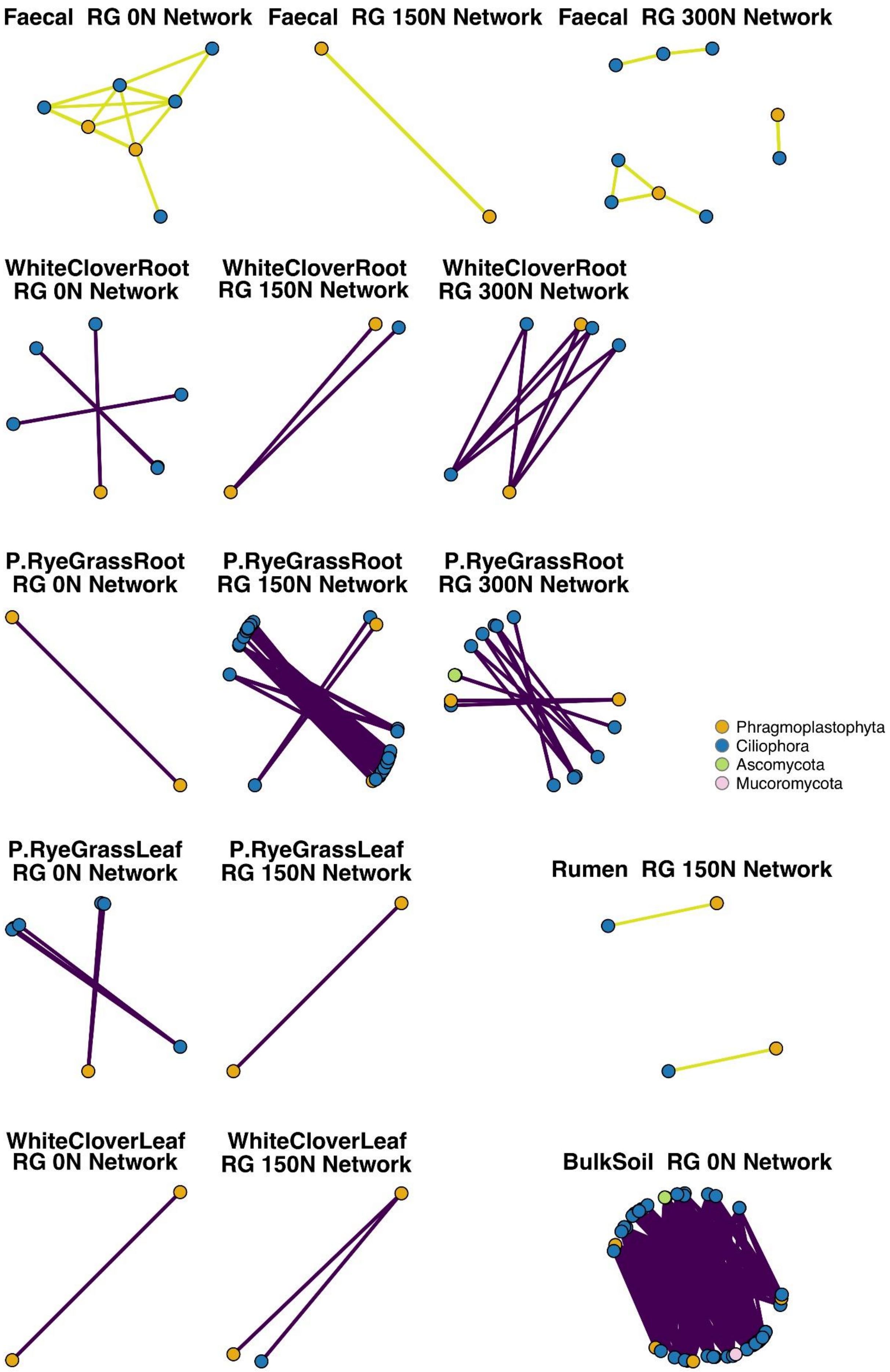

Supplementary Figure S13

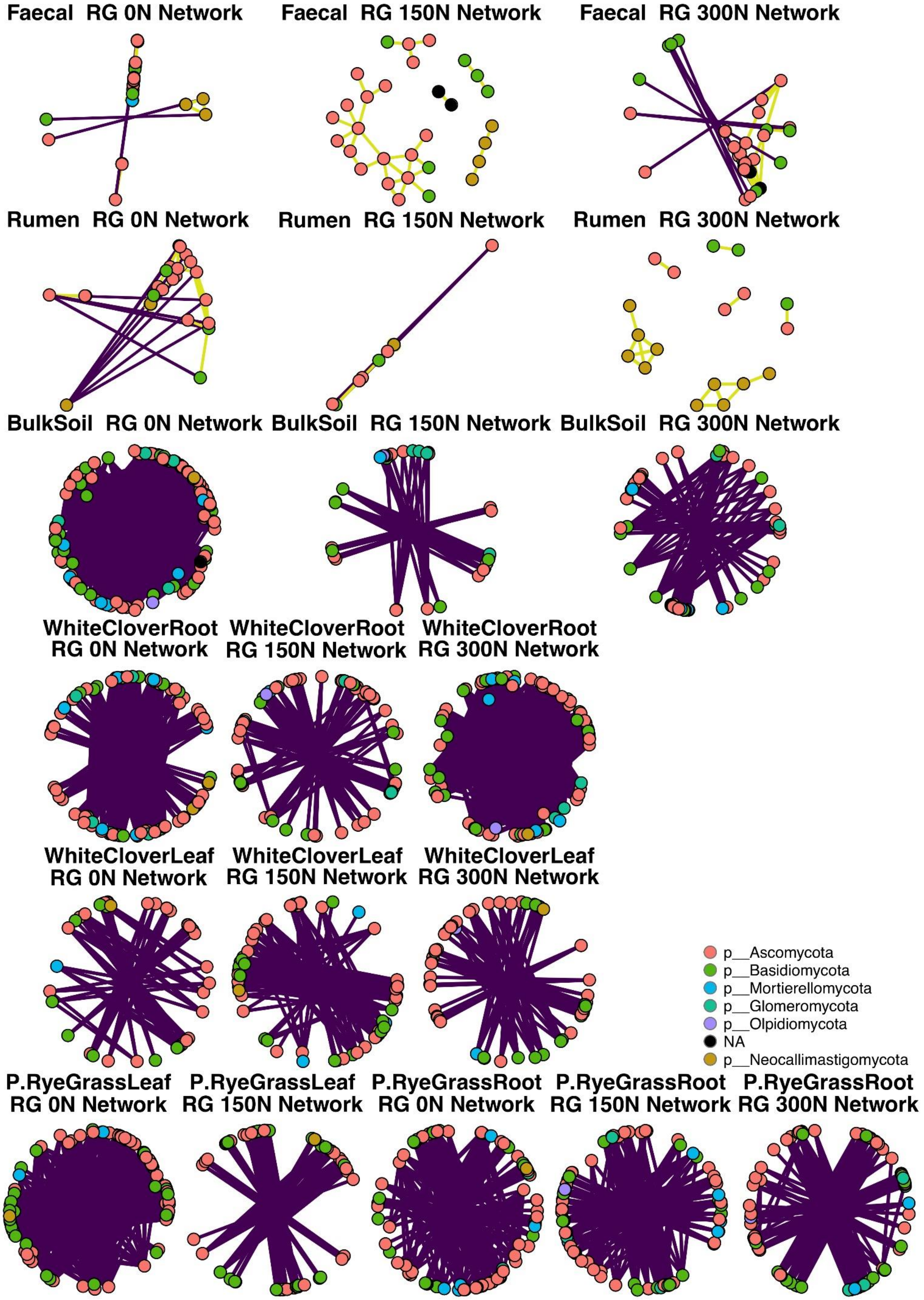

Supplementary Figure S14
